# Novel Cereblon‐Binding Immunomodulators Have Increased Potency Against Gammaherpesvirus‐ Associated Lymphomas In Vitro

**DOI:** 10.1002/jmv.70537

**Published:** 2025-08-06

**Authors:** Prabha Shrestha, Emma N. Treco, David A. Davis, Robert Yarchoan

**Affiliations:** ^1^ HIV and AIDS Malignancy Branch, Center for Cancer Research National Cancer Institute Bethesda Maryland USA

**Keywords:** Burkitt lymphoma, cereblon, Epstein‐Barr virus, human herpesvirus‐8, immunomodulator, Kaposi sarcoma herpesvirus, non‐Hodgkin's lymphoma, primary effusion lymphoma

## Abstract

Primary effusion lymphoma (PEL), which is caused by Kaposi sarcoma herpesvirus (KSHV), and Burkitt lymphoma (BL), a subset of which are associated with Epstein‐Barr virus (EBV), are aggressive non‐Hodgkin's lymphomas. Both have relatively poor survival compared to other lymphomas. Cereblon‐binding immunomodulators (CBIs), such as pomalidomide (Pom), show in vitro efficacy and clinical activity against certain of these lymphomas. Next generation CBIs, such as golcadomide (Golc) and iberdomide (Iber), have increased affinity to the primary cellular target, cereblon, making them potentially better anticancer agents. Here, we report the in vitro activity of these novel CBIs against PEL and BL cell lines. Both Golc and Iber, but primarily Golc, caused substantial growth suppression of PEL and BL lines with much lower half‐maximal inhibitory concentration (IC_50_) compared to Pom. This growth suppression was mediated, in part, by enhanced downregulation of interferon regulatory factor 4 (IRF4) in PEL cell lines. Additionally, both Golc and Iber increased immune surface markers such as ICAM‐1, B7‐2, and MHC‐I in PEL and BL cells at lower concentrations than Pom; these increases led to enhanced recognition of both PEL and BL cells by T‐cells. The novel CBIs had relatively little activity in Pom‐resistant cell lines with low levels of cereblon, suggesting that binding to cereblon is also important for the functions of the novel CBIs. These data show that the newer CBIs are more potent and effective against PEL and BL lines than Pom, and therefore, are worth investigating clinically in patients with these tumors.

## 1 Introduction

Kaposi sarcoma herpesvirus (KSHV), also called human herpesvirus‐8 (HHV‐8), and Epstein‐Barr virus (EBV), also called HHV‐4, are human gammaherpesviruses that establish lifelong infection and can cause several cancers. One such cancer is primary effusion lymphoma (PEL), a rare and aggressive non‐Hodgkin's B‐cell lymphoma (NHL) caused by KSHV; 60%–90% of PEL tumors are coinfected by EBV [1]. PEL predominantly affects immunosuppressed people living with HIV (PLWH) and is notable for its tendency to develop as effusions in the pleural and other body cavities. Patients with PEL are typically treated with an intensive chemotherapy regimen, but even with optimal therapy, survival is relatively poor compared to other lymphomas [2, 3]. BL, another aggressive NHL, is associated with EBV in 30%–40% of cases in PLWH and over 95% of cases in areas where BL is endemic [4]. BL often responds to intensive chemotherapy regimens, though subgroups of BL have a relatively poor prognosis [5]. Hence, it is vital to develop new, more effective therapies for these lymphomas.

We and others previously reported that pomalidomide (Pom) and lenalidomide (Len), two cereblon‐binding immunomodulators (CBIs), have activity against PEL and BL in vitro [6, 7, 8, 9]. Pom and Len function by binding to cereblon, a cellular E3‐ubiquitin ligase, and altering its substrate specificity. This causes the recruitment and subsequent degradation of neosubstrates such as Ikaros (IKZF1), Aiolos (IKZF3), and casein kinase 1 alpha (CK1α), as well as the decrease of cellular oncogenes such as cMyc and interferon regulatory factor 4 (IRF4) [8, 9, 10]. In PEL and EBV‐infected BL cells, Pom has also been shown to upregulate the immune surface markers ICAM‐1 (CD54), and B7‐2 (CD86) during viral latency [7, 11]. Moreover, Pom can upregulate MHC‐I in latent and lytic EBV‐infected cells and can prevent the downregulation of MHC‐1 in lytic PEL cells [7, 11]. These activities require cereblon and occur at least partly through the PI3K/AKT pathway [12, 13]. By increasing immune surface markers on PEL and BL cells, Pom can enhance their recognition and targeting by NK‐cells and T‐cells [7, 13].

Pom is approved by the United States Food and Drug Administration (FDA) for use in multiple myeloma (MM) and was recently approved for Kaposi sarcoma (KS), a KSHV‐associated endothelial cell cancer [14, 15, 16, 17]. Several groups have also reported on the activities of Pom and Len in PEL patients. A 2014 case study described one patient with PEL who achieved complete remission after 18 months of Len treatment [18]. Similarly, Len with DA‐EPOCH and rituximab showed promising results in a trial of PEL patients [3]. In another report, three patients with leptomeningeal PEL (a manifestation that is difficult to treat) were successfully treated with Pom and pembrolizumab [19]. Overall, these results suggest that these and other CBIs may be worth exploring further in PEL and BL.

Next‐generation CBIs have been shown to have increased binding affinity to cereblon. Two of these drugs, golcadomide (Golc), also called CC‐99282, and iberdomide (Iber), also called CC‐220, not only bind with higher affinity to cereblon, but also convert cereblon from an inactive/closed conformation to an active/open conformation more efficiently than Pom or Len [20, 21]. Consequently, Golc and Iber are more effective at degrading the cereblon‐binding neosubstrates Ikaros and Aiolos [22, 23, 24]. Although all CBIs share some neosubstrates, there is evidence that the novel CBIs have a different spectrum of neosubstrate degradation [25]. Golc and Iber have shown enhanced tumoricidal activity in in vitro and in vivo models of various hematological malignancies [26, 27]. Though neither is FDA‐approved, both Golc and Iber are under clinical investigation for use in various conditions. Currently, Golc is being tested in several ongoing trials for various NHLs [28]. It has shown especially promising results in relapsed and/or refractory NHL, as well as previously untreated aggressive B‐cell lymphomas [21, 29]. Similarly, Iber is being tested in clinical trials for the treatment of MM, certain NHLs, and certain autoimmune conditions [28, 30, 31].

Given these findings in other NHLs, we investigated the activity of Golc and Iber on PEL and BL‐derived cell lines to test whether these novel CBIs had activity against these malignancies, and if so, whether they would have greater potency than Pom. In particular, we assessed the effect of these CBIs on cell viability and immune surface marker expression, and tested their activity in cereblon‐deficient Pom‐resistant PEL and BL cells.

## 2 Materials and Methods

### 2.1 Cell Culture and Reagents

PEL cell lines BCBL‐1, JSC‐1, BC‐3, BC‐1, and BC‐2, and BL cell lines Raji, Daudi, and CA46 were obtained and maintained as described previously [7, 13]. EBV^+^ and EBV^−^ BL41 cell lines were a kind gift from Bill Sugden (UW‐Madison). The IL2‐Jurkat T‐cell line was obtained from Promega (Madison, WI, cat # J1651) and grown in complete media with 200 µg/mL hygromycin (ThermoFisher Scientific). Pom‐resistant (PomR) BCBL‐1 and Daudi cells were previously generated and cultured as described [12, 13]. Pom, Golc, and Iber were obtained from Selleck Chemical (Houston, TX), and stock solutions of 100 µM were made in dimethylsulfoxide (DMSO) (Sigma).

### 2.2 Viability Assays

Growth and viability of PEL and BL cells were assessed by plating the cells at 5 × 10^4^ cells/mL for 5 days in 96‐well plates in the presence of DMSO control or various concentrations of the CBIs. Relative viability was then measured 5 days posttreatment using CellTiter 96 Aqueous One Solution Cell Proliferation Assay (Promega) according to the manufacturer's protocol. Viability curves were plotted using a sigmoidal 4‐parameter logistic (4‐PL) curve‐fitting model using Graphpad Prism (v10.4.0), and absolute IC_50_ concentrations were calculated from the 4‐PL curves with the baseline set to 0.

### 2.3 Flow Cytometry Assays for Surface Marker Expression

Analysis of surface marker expression was carried out by flow cytometry as described previously [11] using PerCP/Cy5.5‐conjugated antibodies against CD86 (cat # 374215), CD54 (cat # 353119), MHC‐I (cat# 311420), IgG1κ isotype control (cat # 400150) purchased from Biolegend, San Diego, CA, and MICA (FAB1300P) purchased from R&D, Minneapolis, MN. Flow cytometry was performed using Cytoflex S flow cytometer (Beckman Coulter, Indianapolis, Indiana), and data were analyzed using FlowJo software (FlowJo LLC, Ashland, OR). Only live cells were gated based on forward vs. side scatter properties and used for surface marker analysis. Median fluorescence intensity (MFI) obtained for isotype control was subtracted before calculating fold changes in the levels of surface markers in CBI‐treated cells over DMSO control‐treated cells.

### 2.4 Western Blot Analysis

Cells were cultured at 3 × 10^5 ^cells/mL for 48 h in the presence of DMSO control or CBIs. Protein lysates were extracted using NE‐PER Nuclear Extraction kit (ThermoFisher Scientific, Waltham, MA) (where indicated) according to the manufacturer's protocol. Western blot analyses were performed, and images were analyzed using the Odyssey imaging system and ImageStudio software (Li‐Cor) as described previously [13]. A list of primary antibodies used for western blot analysis can be found in Supporting Information: Table S1.

### 2.5 T‐Cell Activation Assay

T‐cell activation assays were performed using Jurkat T‐cells expressing a luciferase reporter gene under interleukin‐2 (IL‐2) promoter (IL‐2 Jurkat cells) (Promega, cat# J1651) as the effector cells. 1 µg/mL anti‐human CD3 monoclonal antibody (OKT3 from ThermoFisher Scientific, cat# 16‐0037‐81) was used to activate the T cells, and PEL or BL cell lines pretreated for 2 days with DMSO control or CBIs were used to co‐stimulate them. The assay was carried out as described previously [13].

### 2.6 Statistical Analysis

Statistical analysis was performed in Microsoft Excel using unpaired, two‐tailed, Student's *t*‐test on experiments with at least three biological replicates. No correction was made for multiple tests. *p* values less than or equal to 0.05 were considered statistically significant.

## 3 Results

### 3.1 Novel CBIs Are More Potent Than Pom in Reducing the Viability of PEL Cell Lines

**Figure 1:**
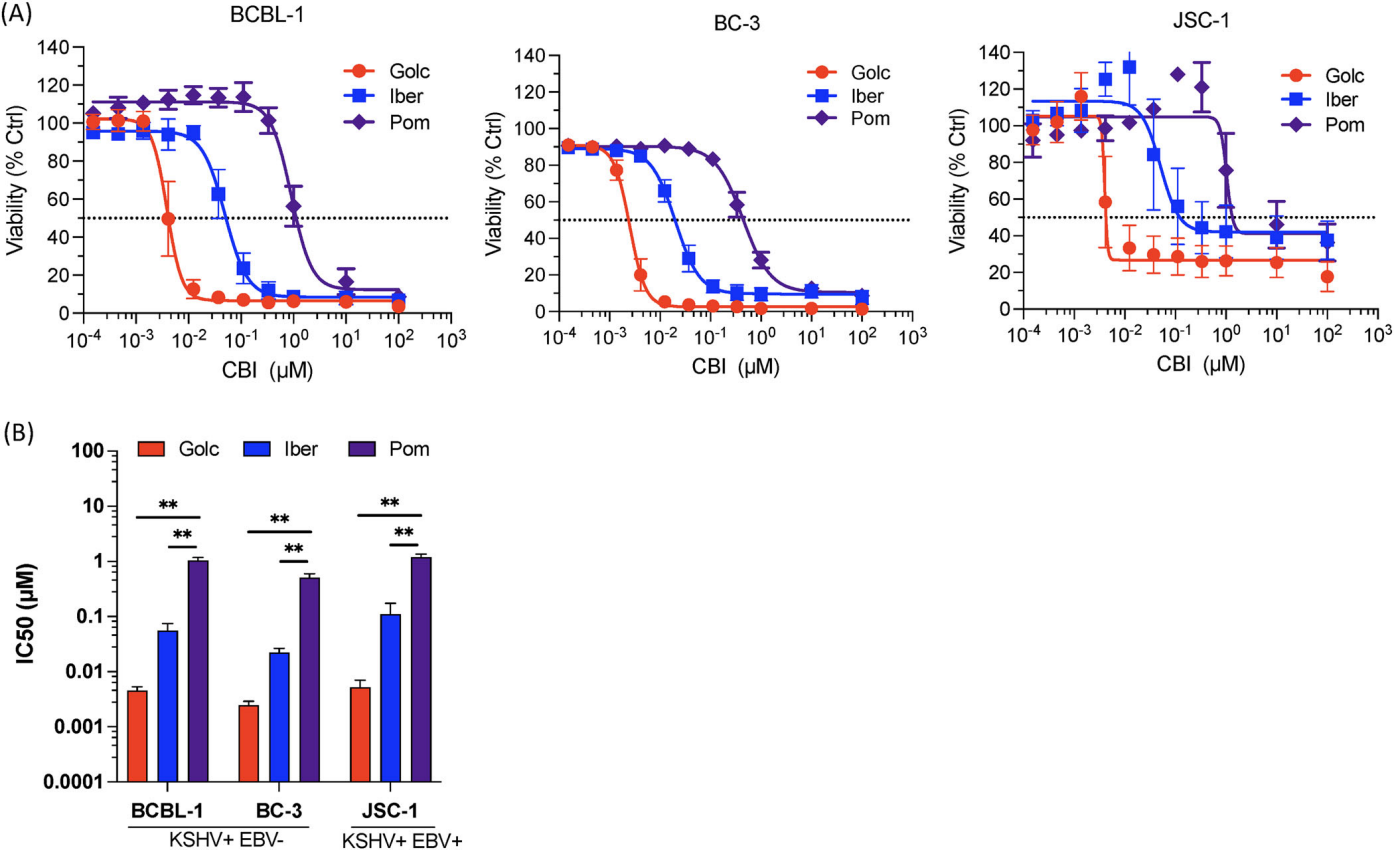
Viability of PEL cell lines when cultured with CBIs. (A) KSHV‐positive/EBV‐negative (BCBL‐1 and BC‐3) and KSHV/EBV‐positive (JSC‐1) PEL cell lines were cultured in the presence of DMSO control or various concentrations of Golc, Iber, or Pom for 5 days. Cell viability was measured using the ATP viability assay (Promega), and relative viability in the presence of CBIs was calculated as a percentage of DMSO control. Error bars represent the standard deviations from three separate experiments, and dose–response curves were plotted using 4PL best fit graphs. Dotted lines represent 50 percent growth inhibition. (B) Absolute IC_50_ values for growth inhibition by each of the CBI in each of the PEL cell line are shown. IC_50_ determinations were made separately from each of the three separate experiments; shown are the mean and standard deviation of these IC_50_ values. Statistically significant differences in IC_50_ are shown. **p ≤ 0.01, unpaired, two‐tailed, *t*‐test.

The effects of Golc, Iber, and Pom on the viability of 5 PEL cell lines were assessed using an ATP viability assay (Figure 1A and Supporting Information: Figure S1A). While all three CBIs had activity in each line, Golc and Iber were substantially more potent than Pom (Figure 1A,B and Supporting Information: Figure S1A,B). Notably, IC_50_ values for Golc were lower than its reported Cmax (around 0.04–0.05 µM) [21] in both EBV^−^ as well as in EBV^+^ PEL cell lines, except for BC‐1, where it was approximately the Cmax. By contrast, the IC_50_ values for Iber and Pom were higher than their respective Cmax values of 0.01 and 0.3 µM (Figure 1B and Supporting Information: Figure S1B) [17, 32].

### 3.2 Burkitt Lymphoma Cell Lines Respond Variably to CBIs

**Figure 2:**
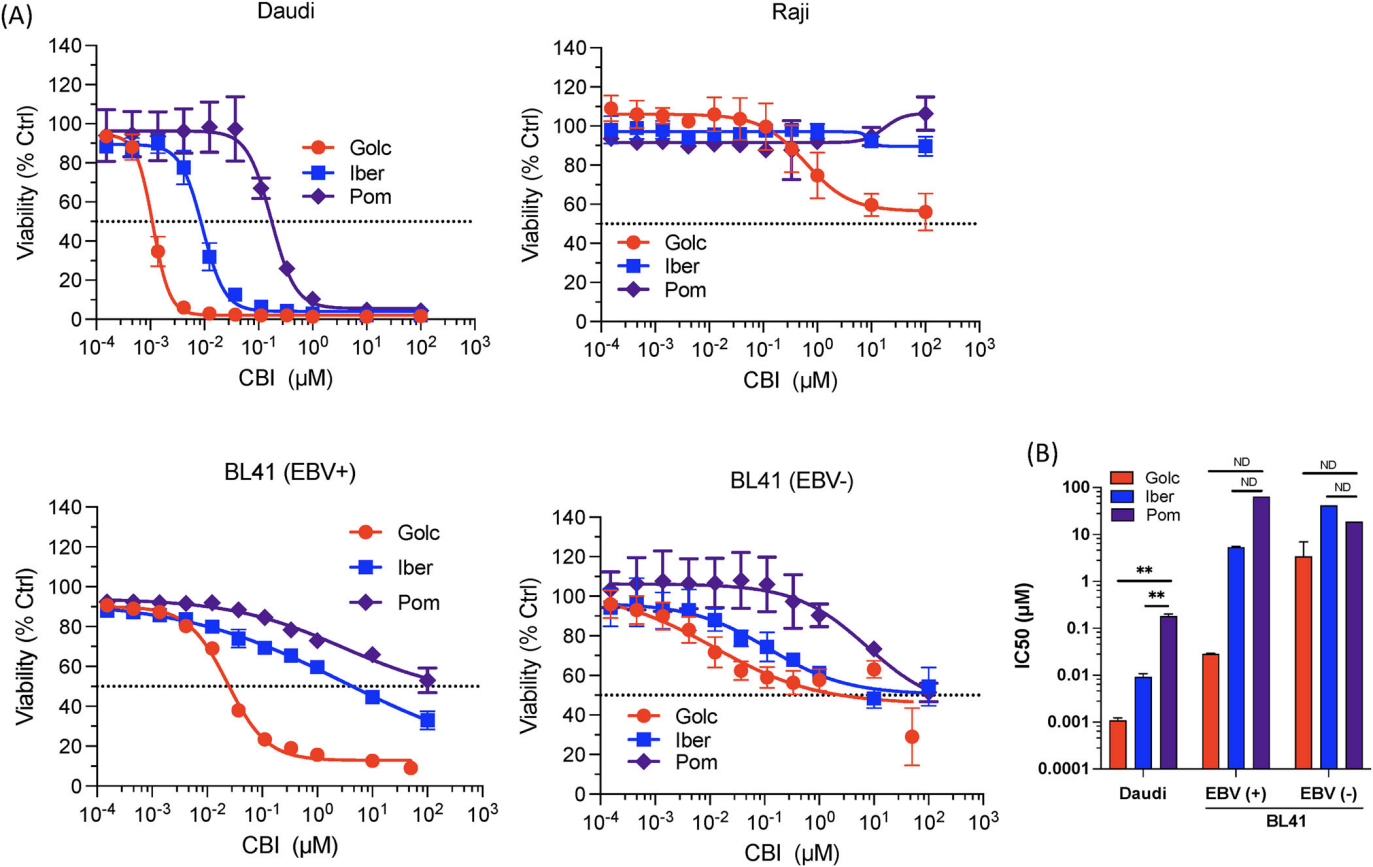
Viability of BL cell lines with CBIs. (A) EBV(+) BL cell lines, Daudi and Raji, as well as EBV(+) and EBV(−) BL41 BL cell lines were cultured in the presence of DMSO control or various concentrations of Golc, Iber, or Pom for 5 days. Cell viability was measured using ATP viability assay (Promega), and relative viability in the presence of CBIs was calculated as a percentage of DMSO control; dose–response curves were plotted using 4PL best fit graphs. Data show averages from three biological replicates ± standard deviations. (B) Absolute IC_50_ values for growth inhibition of Daudi and BL41 lines by each of the CBI were calculated separately from each individual experiment and the mean and standard deviations of these IC_50_ values are shown. IC_50_ was not attained with Raji at the concentrations utilized. Statistically significant differences in IC_50_ values are shown. **p ≤ 0.01; unpaired, two‐tailed, *t*‐test. ND *p* values were not determined because IC_50_ for Pom‐treatment could be determined only from one experiment in both BL41 lines and that for Iber could be determined only from two experiments in BL41 EBV(−) line; the rest of the bars represent three experiments.

We next evaluated the effect of Golc, Iber, and Pom on the viability of various BL cell lines and found that these lines respond variably (Figure 2A,B). Daudi (EBV^+^) and EBV^+^ BL41 cells were sensitive to all three CBIs, with Golc showing the greatest growth suppression in these lines. EBV^−^ BL41 cells also responded to all three drugs, although with maximum suppression of only about 50% at the highest concentration tested (100 µM). Raji, a line infected with a mutated form of EBV that cannot produce virus, only responded to Golc and even with Golc had a maximum suppression of less than 50%. In Daudi the IC_50_ values of Golc and Iber were substantially lower than those of Pom; the IC_50_ of Golc in particular was also lower than its reported Cmax (around 0.04–0.05 µM) [21] (Figure 2B). In the EBV^+^ BL41 line, the IC_50_ of these drugs also seemed lower than Pom, although statistical analysis was hampered because the IC_50_ of Pom was above the highest dose tested. By contrast, in the EBV^−^ BL41 line, the IC_50_ of Golc was substantially higher than in EBV^+^ BL lines, and it could not be consistently achieved for Iber and Pom (Figure 2B). Altogether, these data suggest that only some BL lines are responsive to the old CBIs, and that they all generally respond to Golc to some degree.

### 3.3 Effects of the CBIs on Cellular and Viral Genes Are Variable

**Figure 3:**
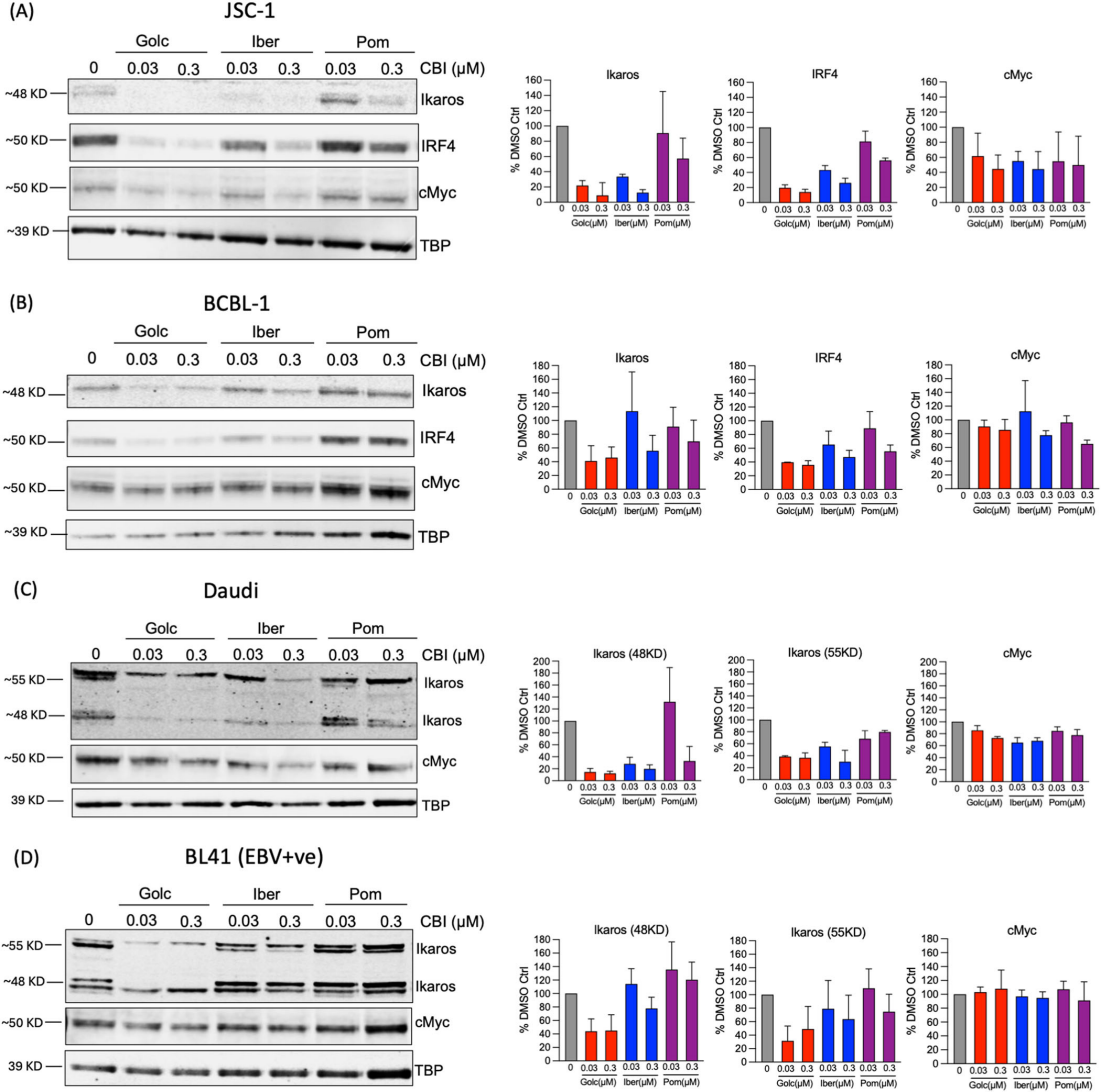
Effects of CBIs on selected downstream target proteins in PEL and BL cell lines. PEL cell lines JSC‐1 (A) and BCBL‐1 (B) as well as EBV( + ) BL cell lines Daudi (C) and BL41 (EBV+ve) (D) were cultured for 2 days with DMSO control or indicated concentrations of Golc, Iber, or Pom. Levels of Ikaros, IRF4, and cMyc were measured by western blot analysis of the nuclear lysates. The experiment was done three times (except for Daudi, which was done twice), and representative blots are shown. Bar diagrams represent average levels of Ikaros, IRF4, and cMyc in CBI‐treated cells as a percent of DMSO‐treated cells after normalizing for the level of TBP (loading control); error bars represent standard deviations. *Note:* IRF4 was nondetectable in BL cell lines.

CBI‐induced decreases in IRF4 and cMyc were previously reported to play an important role in the growth suppression of PEL cells [8, 9]. Ikaros is directly degraded by the cereblon E3‐ubiquitin ligase complex in the presence of CBIs and provides another marker for CBI activity, although it is not essential for PEL cell viability [9]. Based on western blots, while all 3 CBIs led to some decrease in Ikaros and IRF4 levels, 0.3 µM and 0.03 µM Golc led to more substantial decreases in these proteins compared to treatment with similar concentrations of Pom in both PEL cell lines (Figure 3A,B). Interestingly, the level of cMyc downregulation was about the same with all three CBIs (Figure 3A,B). Similarly, Golc and Iber led to a greater decrease in Ikaros than Pom in BL cell line Daudi (note: multiple isoforms of Ikaros are observed in BL lines as indicated in Figure 3C,D), while only Golc was substantially better at decreasing Ikaros in the BL41^+^ line, consistent with viability data in Figure 2. IRF4 was not detected in either BL cell lines (data not shown), consistent with previous reports that IRF4 is expressed at very low/undetectable levels in BL cells [33, 34]. cMyc is translocated to an IgG locus in BL cells; nevertheless, we measured its level but found no substantial change in its level with the CBIs. These data indicate that a more substantial IRF4 downregulation might mediate increased activity of the new CBIs, particularly Golc, in PEL cell lines but not BL cell lines.

We next determined the effect of the CBIs on select viral gene expression by qRT‐PCR (Supporting Information: Figure S2). Genes measured for KSHV were LANA (latent) and RTA (lytic); those for EBV were EBNA‐1 (latent), EBER‐2 (a latent noncoding RNA), and BMRF‐1 (lytic). In the PEL cell lines, both Iber and Pom showed a trend towards a decrease in both latent and lytic KSHV and EBV genes (Supporting Information: Figure S2A). Surprisingly, this effect was not seen with Golc, suggesting that the changes in viral genes are likely not associated with the increased potency of novel CBIs. The BL cell line Daudi did not show any consistent change in EBV genes, while BL41^+^ line showed a trend towards decrease in EBNA‐1 and BMRF‐1 (EBER‐2 was undetectable in this line) with Golc and Iber but not Pom (Supporting Information: Figure S2B), again showing that the BL lines respond variably to the CBIs.

### 3.4 Pom‐Resistant PEL and BL Cell Lines Do Not Respond to Golc or Iber

**Figure 4:**
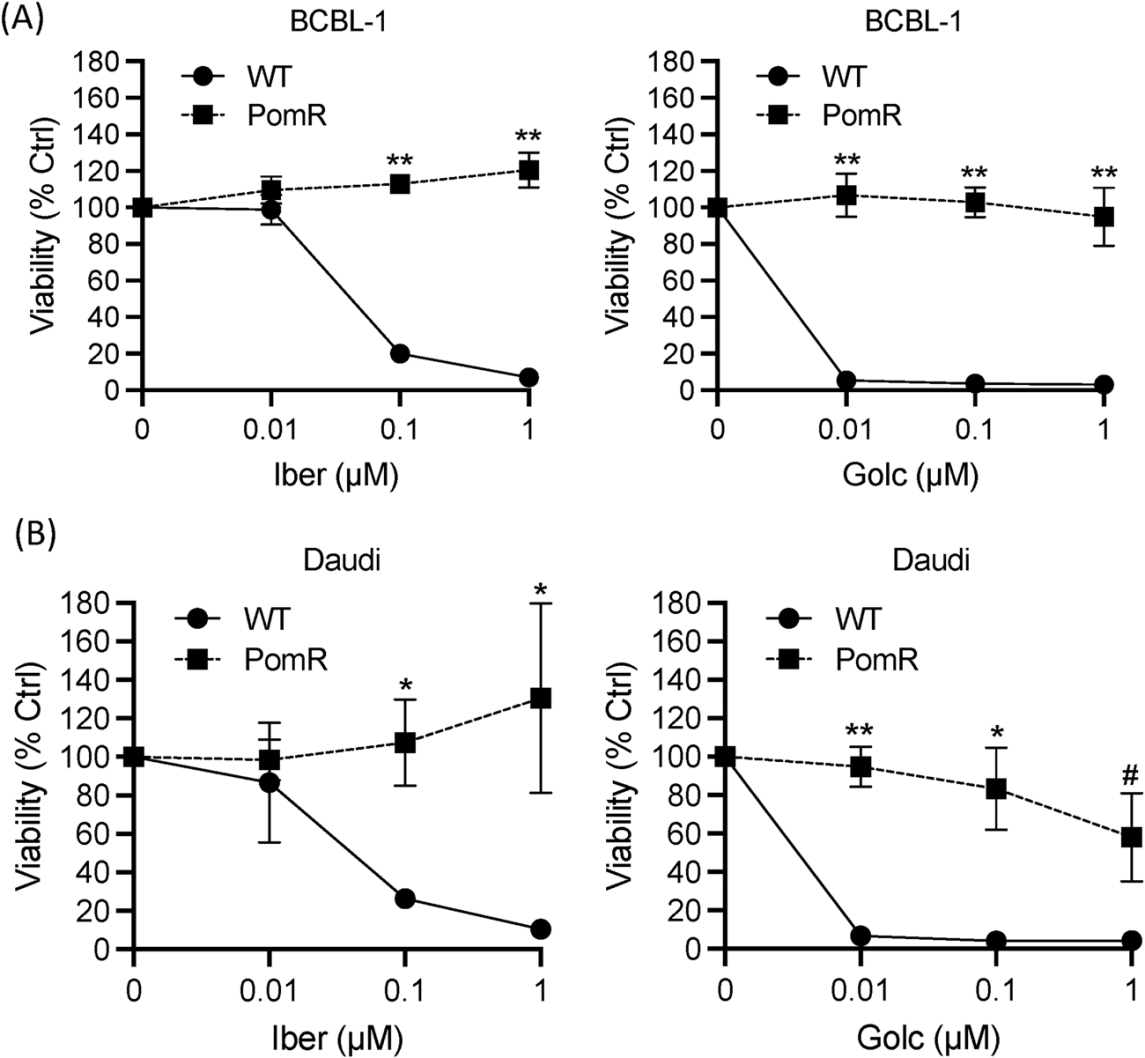
Effects of Golc and Iber on the viability of Pom‐resistant cells. (A and B) Parental (WT) or Pom‐resistant (PomR) BCBL‐1 (A) or Daudi (B) cells were treated with DMSO control or indicated concentrations of Iber or Golc for 5 days. Cell viability was measured using an ATP viability assay (Promega), and relative viability in the presence of CBIs was calculated and plotted as a percentage of DMSO control. Data show averages from three biological replicates (except four replicates for BCBL‐1 with Golc) and error bars represent standard deviations. Statistically significant differences (*p ≤ 0.05; **p ≤ 0.01; unpaired, two‐tailed, *t*‐test) between parental and PomR cells at the same concentration of CBI are indicated. ^#^ indicates *p* = 0.06.

Patients with MM can develop resistance to Pom, primarily via downregulation of cereblon (reviewed in [25]). Patients that are resistant or refractory to an older CBI like Len and Pom can still show favorable response to a newer CBI like Iber [30, 31]. Thus, we wanted to explore whether Golc and Iber would still be effective in PEL and BL cells that were resistant to Pom. To this end, we used Pom‐resistant (PomR) BCBL‐1 and Daudi cell lines that were previously generated by exposing the cells to increasing concentrations of Pom [12, 13]. These cells expressed about 20%–40% of the normal levels of cereblon in the wild‐type (WT) cells and did not show decreases in viability with Pom [12, 13] (Supporting Information: Figure S3A,B). The viability of PomR BCBL‐1 and PomR Daudi cells was also no longer reduced by Iber at concentrations up to 1 µM (Figure 4A,B). Similarly, Golc did not decrease the viability of PomR BCBL‐1 cells, although it did lead to a moderate decrease in the viability of PomR Daudi cells, especially at 1 µM.

### 3.5 Golc and Iber Increase Immune Surface Markers on PEL and BL Cell Lines

**Figure 5:**
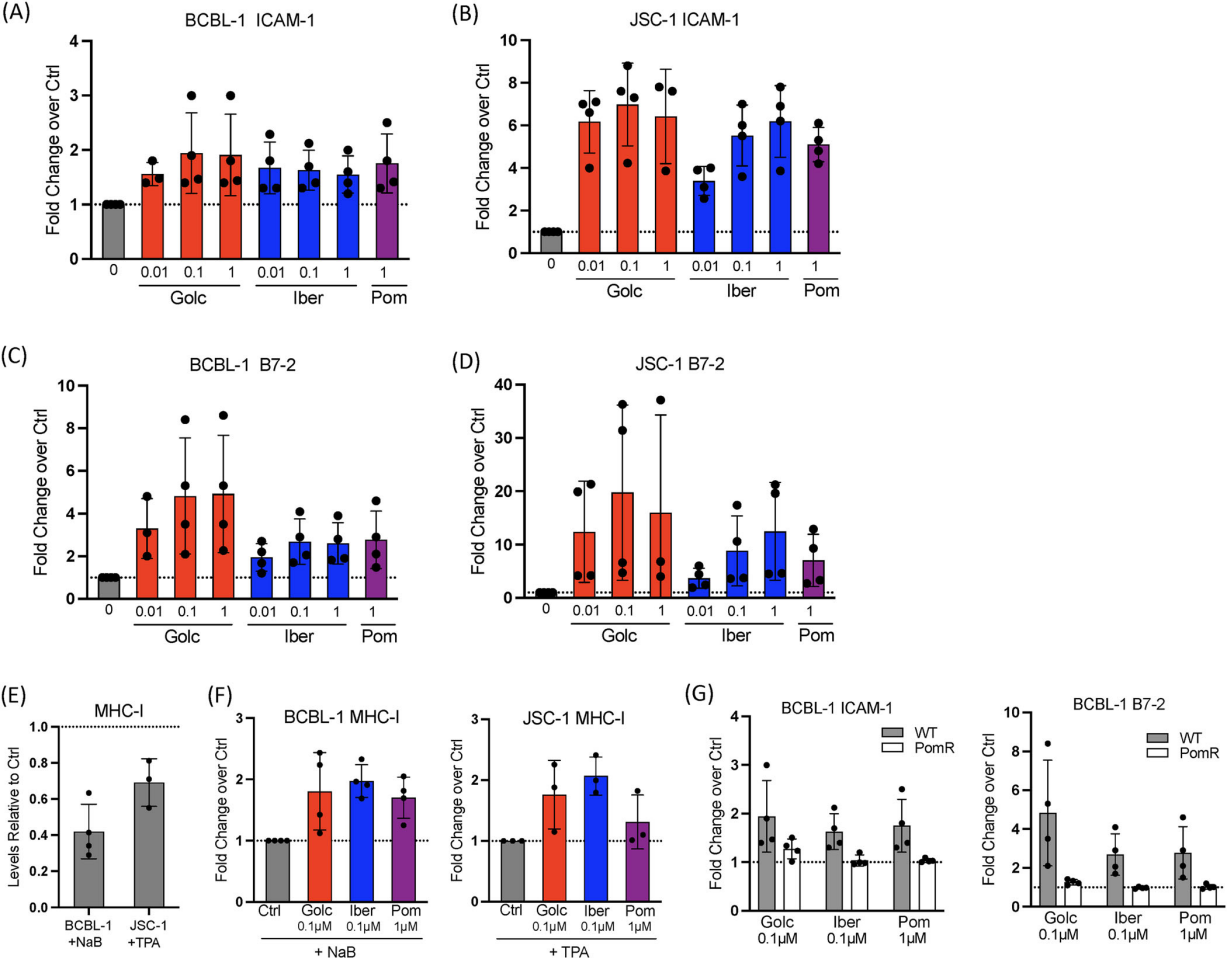
Golc and Iber increase immune surface markers in PEL cells. Cell surface markers on PEL cell lines, BCBL‐1 and JSC‐1, were measured by flow cytometry. Median fluorescent intensity (MFI) obtained for isotype control was subtracted and fold changes over DMSO control were calculated. Dots represent fold changes from each experiment. (A–D) Changes in the levels of ICAM‐1 (A and B) and B7‐2 (C and D) in BCBL‐1 and JSC‐1 cells, respectively 2 days after treatment with CBIs. Shown are averages and standard deviations of fold changes from four independent experiments. Dots represent fold changes from each experiment. (E–F) Surface MHC‐I levels were measured after lytic induction of BCBL‐1 and JSC‐1 cells with NaB (BCBL‐1) or TPA (JSC‐1). Cells were pretreated with DMSO control or CBIs for 24 h (BCBL‐1) or 48 h (JSC‐1) and then treated with NaB or TPA for another 24 h before measuring surface MHC‐I levels. (E) Expression of MHCI compared to control by NaB or TPA treatment alone of BCBL‐1 and JSC‐1 cells, respectively. (F) Fold change in MHC‐I levels by CBIs relative to DMSO control in NaB (BCBL‐1) or TPA (JSC‐1) treated cells. Shown are average fold changes and standard deviations from four (BCBL‐1) or 3 (JSC‐1) independent experiments. (G) Average fold changes and standard deviations from 3 experiments in the levels of ICAM‐1 and B7‐2 in wild‐type (WT) or Pom‐resistant (PomR) BCBL‐1 cell lines after treatment with indicated concentrations of CBIs.

In PEL and BL cell lines, Pom was previously shown to increase immune surface markers ICAM‐1 and B7‐2 during latency [11, 13]. It was also shown to prevent the downregulation of MHC‐I during KSHV lytic reactivation in PEL cell lines. We examined how Golc and Iber would affect these markers in PEL and BL cells 2 days posttreatment with CBIs when cell viability is mostly unaffected (Supporting Information: Figure S4). Flow cytometry analysis of PEL cell lines, BCBL‐1 and JSC‐1, revealed that, like Pom, Golc and Iber also showed a trend towards increases in ICAM‐1 and B7‐2, but did so at lower concentrations than Pom (Figure 5A–D and Supporting Information: Figure S5A–C). Increases in these markers were also observed in the cell extracts by western blot analysis (Supporting Information: Figure S5C). As expected, none of the CBIs substantially increased MHC‐I in latent PEL cell lines (Supporting Information: Figure S6A,B). Lytic reactivation of KSHV in PEL cell lines, induced by NaB or TPA, led to decreases in surface MHC‐I levels (Figure 5E and Supporting Information: Figure S6C). This decrease could be prevented, to some degree, by all three CBIs (0.1 µM Golc or Iber and 1 µM Pom) in both BCBL‐1 and JSC‐1 cells (Figure 5F and Supporting Information: Figure S6C). Next, ICAM‐1 and B7‐2 levels were measured in PomR BCBL‐1 cells. Like Pom, 0.1 µM Golc and Iber could not increase ICAM‐1 and B7‐2 in PomR cells (Figure 5G), suggesting that the increase in these markers by Golc and Iber could be, at least in part, mediated through cereblon.

**Figure 6:**
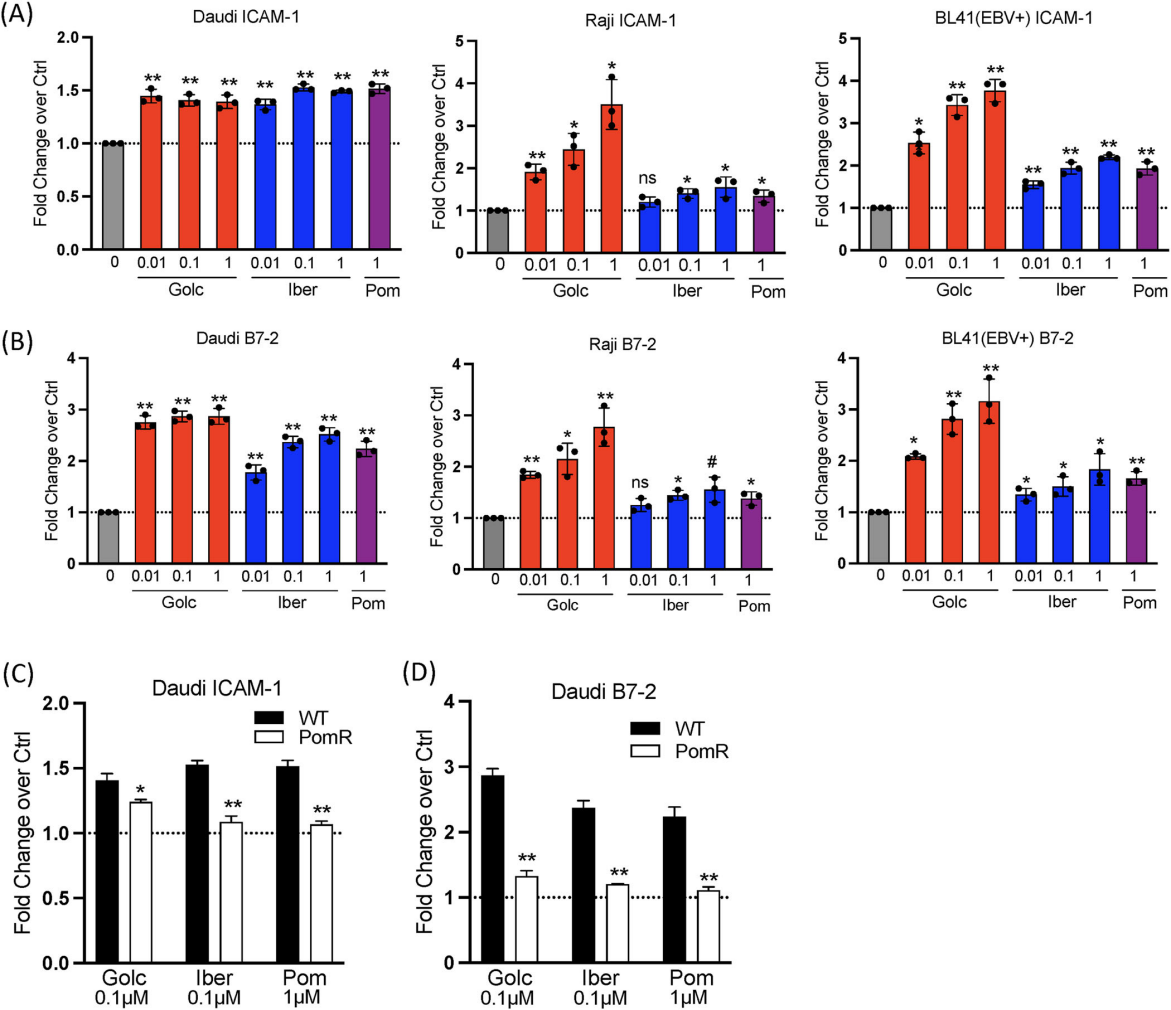
Golc and Iber increase immune surface markers in BL cells. BL cell lines were cultured for 2 days with DMSO control or CBIs, and levels of immune surface markers were measured by flow cytometry. Median fluorescent intensity (MFI) obtained for isotype control was subtracted and fold changes over DMSO control were calculated. Shown are averages from three independent experiments ± standard deviations. (A and B) Levels of ICAM‐1 (A) and B7‐2 (B) in Daudi, Raji, and BL41(EBV+) cell lines. Statistically significant differences between DMSO control and CBIs are shown. *p ≤ 0.05; **p ≤ 0.01; ^#^ *p* = 0.06; unpaired, two‐tailed, *t*‐test. (C and D) Levels of ICAM‐1 (C) and B7‐2 (D) in wild‐type (WT) or Pom‐resistant (PomR) Daudi cell lines after treatment with indicated concentrations of CBIs. Fold changes between DMSO control and CBI‐treatment were calculated and statistically significant differences in CBI‐induced increases between WT cells and PomR cells are shown. *p ≤ 0.05; **p ≤ 0.01; unpaired, two‐tailed, *t*‐test.

In EBV‐infected BL cell lines, all three CBIs led to significant increases in ICAM‐1 and B7‐2; these increases were somewhat more pronounced and occurred at relatively lower concentrations of Golc than Iber in the Raji and EBV^+^ BL41 lines (Figure 6A,B and Supporting Information: Figure S7A). Notably, while Golc and Iber showed substantial effects at 0.01 and 0.1 µM, Pom did so only around 1 µM (Figure 6A,B and Supporting Information: Figure S7B). Western blot analyses of the extracts from BL cell lines also showed that increases in surface markers occurred with all three drugs (Supporting Information: Figure S7C). An additional surface marker, MHC class I chain‐related gene A (MICA), also showed increases with the CBIs in Daudi but minimally or not at all in Raji cells (Supporting Information: Figure S8). Unlike the EBV^+^ BL lines, EBV^−^ BL41 and CA46 cells either showed only a minor increase (B7‐2 in EBV^−^ BL41) or no change in ICAM‐1 or B7‐2 with the CBIs (Supporting Information: Figure S9A,B). Also, like PomR BCBL‐1 cells, PomR Daudi cells showed less substantial increases in ICAM‐1 and B7‐2 compared to WT cells in response to the CBIs, although 0.1 µM Golc still led to a small but significant increase in ICAM‐1 (*p* value of 0.002 by two‐tailed *t*‐test) (Figure 6C,D). Together, these data suggest these novel CBIs also have minimal activity in cereblon‐lacking PomR cells, but that even in these cells, Golc has some activity.

### 3.6 Golc and Iber Enhance the Ability of PEL and BL Cell Lines to Activate T Cells

**Figure 7:**
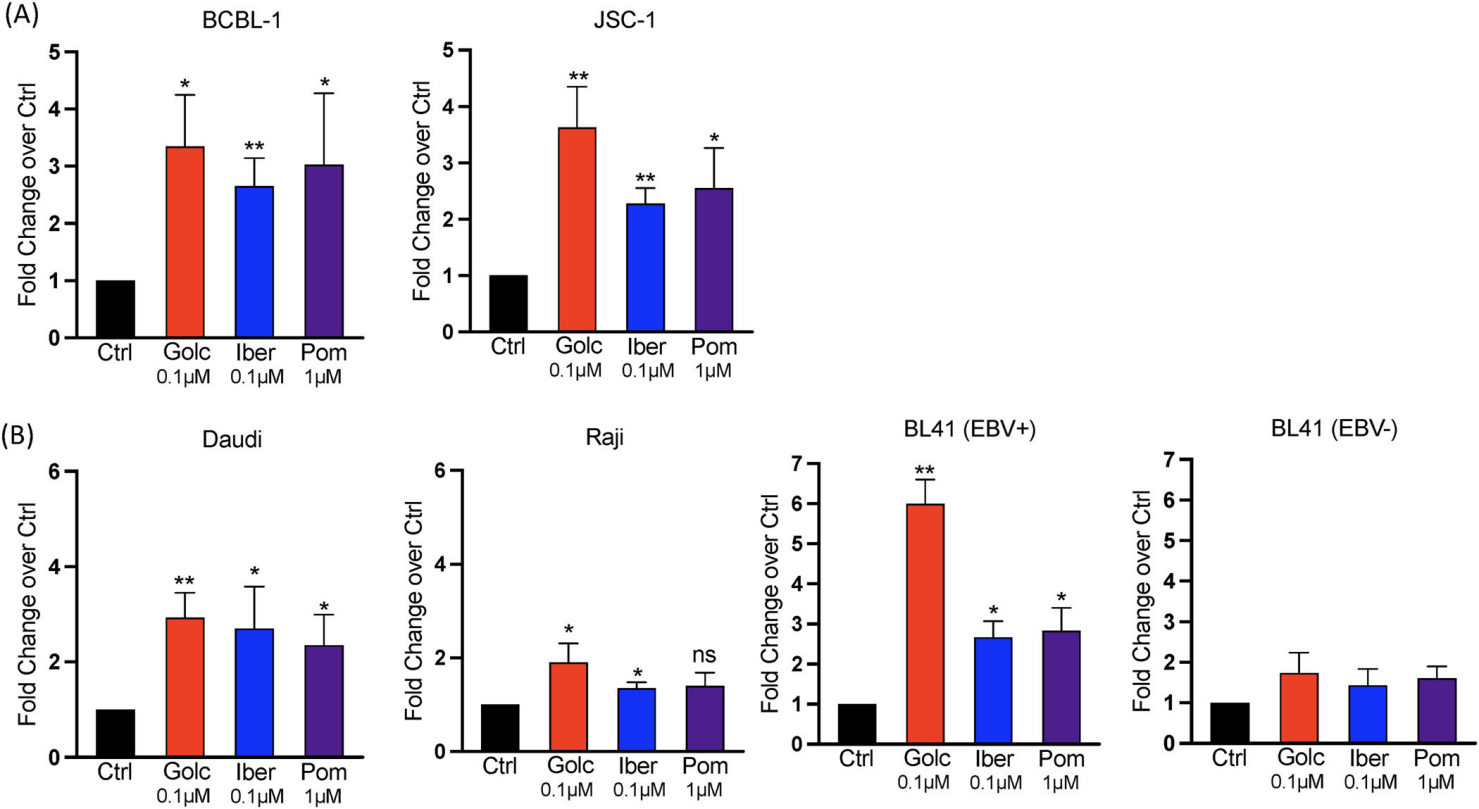
Increased T‐cell activation following CBI‐treatment of PEL and BL cell lines. PEL cell lines (A) and BL cell lines (B) were cultured for 2 days with DMSO control or indicated concentrations of CBIs, and then co‐incubated for 6 h with IL2‐Jurkat cells at a 2:1 (PEL cells) or 1:5 (BL cells) target‐to‐effector ratio in the presence of 5 µg/mL anti‐human CD3 antibody to measure Jurkat T‐cell activation. Data shows average fold changes in relative light units from IL2‐Jurkat cells co‐incubated with CBI‐treated target cells over those co‐incubated with DMSO‐ctrl treated cells. Shown are averages from four independent experiments ± standard deviations. Statistically significant differences (*p ≤ 0.05; **p ≤ 0.01; unpaired, two‐tailed, *t*‐test) in fold changes between DMSO control and CBI‐treated cells are shown.

ICAM‐1 and B7‐2 bind to their receptors LFA‐1 and CD28, respectively on T cells, and provide costimulatory signals for their activation. Pom‐induced increases in these markers were previously shown to contribute to an enhancement in T‐cell activity against PEL and BL cell lines [7, 13]. To assess T‐cell activation against PEL and BL cells treated with the novel CBIs, we performed T‐cell activation assays using IL‐2 Jurkat T‐cells as effector cells. PEL cell lines (BCBL‐1 and JSC‐1) and BL cell lines (Daudi, Raji, and BL41) were pretreated for 2 days with DMSO control or the CBIs and then co‐incubated for 6 h with IL‐2 Jurkat cells that were activated with OKT3 anti‐human CD3 antibody. BCBL‐1 and JSC‐1 PEL lines treated with each of the three CBIs induced significant increases in T‐cell activation (Figure 7A). As for BL, only the EBV^+^ lines (Daudi, Raji, and EBV^+^ BL41) induced significant increases in T‐cell activation upon treatment with 0.1 µM of Golc or Iber (Figure 7B). Treatment with 0.1 µM Golc induced equivalent or higher increases than either 0.1 µM Iber or 1 µM Pom; this is most evident in the EBV^+^ BL41 line (Figure 7B). These data suggest Golc‐ and Iber‐induced increases in ICAM‐1 and B7‐2 on PEL and EBV^+^ BL cells lead to enhanced costimulation of T‐cell activation.

## 4 Discussion

The initially developed cereblon‐binding immunomodulators thalidomide, Len, and Pom have already shown activity in the treatment of various malignancies, including multiple myeloma, Kaposi sarcoma, and certain NHL. Here, we show that two novel CBIs, Golc and Iber, are more potent than Pom at decreasing the growth and viability of most of the PEL and BL‐derived cell lines tested. Our findings show that while all 3 CBIs decrease the viability of most PEL and BL cell lines, Golc does so at IC_50_ values well under the reported Cmax [21] in the majority of the cell lines tested. This is in contrast to Pom, which is clinically effective but exerts direct cytotoxic effects on PEL and BL cells in vitro only at doses higher than the clinical Cmax. Taken together, these results make Golc a particularly attractive CBI for further clinical testing in these malignancies.

IRF4 is overexpressed in and is essential for the survival of PEL cell lines [35], and a previous report showed that Pom's effects on PEL cell viability could be partially rescued upon exogenous IRF4 expression [9]. Thus, the higher potency of Golc in PEL cells could be, at least partially, due to its enhanced ability to decrease IRF4. However, somewhat surprisingly, decrease in IRF4 did not correlate with downregulation of cMyc, whose expression is often regulated by IRF4. It is thus likely that the novel CBIs induce additional/unique changes that could mediate their observed effects in PEL cells; this possibility remains to be explored.

Unlike PEL cell lines, BL cell lines showed variable responses to the CBIs, with some showing a strong response to all 3 CBIs while some only responding to Golc. BL cells express little to no IRF4, and the expression of cMyc, which is regulated by noncanonical regulatory regions due to its translocation in BL [33, 34], was not affected by the CBIs; thus these proteins are likely not involved in the CBI‐induced growth suppression of BL cells. It is also noteworthy that among the BL lines, those that are EBV^+^ tended to have greater CBI‐induced growth suppression. While we did not directly explore the mechanisms behind this observation, previous literature on the use of CBIs on BL cell lines potentially provide some clues. The EBV genes, EBV‐encoded small RNA 1 (EBER1) and latent membrane protein 1 (LMP1), have been shown to promote cell growth by directly suppressing the levels and activity of p21 tumor suppressor in various models [36, 37]. Interestingly, Pom and Len were shown previously to increase p21 levels and inhibit CDK activity in BL cell lines [6, 38]. Thus, the enhanced effect of the novel CBIs on the viability of BL cells, especially the ones positive for EBV, could be due to greater changes in p21 and CDK activities. In another study, Len and Pom were shown to induce lytic reactivation of EBV in BL cell lines [39]. While we did not observe increases in the mRNA levels of the EBV genes with any of the CBIs, it is still possible that the regulation of these genes and, consequently, lytic reactivation of EBV by the CBIs, might occur downstream of mRNA expression. Hence, lysis of lytically induced BL cells could also, at least partially, account for the effects of the novel CBIs on the viability of EBV^+^ BL cell lines. Alternatively, changes in yet unidentified novel targets could also be responsible for the observed effects in BL cell lines. Understanding these mechanistic possibilities warrant further studies.

In addition to suppressing growth, our study shows that the novel CBIs can also increase immune markers on the surface of PEL and BL cell lines, leading to their higher recognition by T‐cells, at much lower concentrations compared to Pom. Here, we focused primarily on ICAM‐1 and B7‐2 because our previous study showed that most of the increase in T‐cell activation by Pom‐treated PEL cell lines was due to increases in surface ICAM‐1 and B7‐2 [13]. Neutralizing either of these markers in Pom‐treated PEL cells led to almost complete prevention of T‐cell activation [13]. Nevertheless, it is possible that the CBIs also lead to changes in other immune markers or secreted factors that could potentially affect T‐cell recognition of the CBI‐treated PEL and BL cells.

The primary cellular target of the CBIs, cereblon, is necessary for the direct target cell activities of the CBIs, and a decrease in cereblon levels is a common mechanism by which tumor cells develop resistance to CBIs [25, 40]. Here, we found that the ability of Golc and Iber to suppress growth and increase immune surface marker expression is reduced in Pom‐resistant cells with decreased levels of cereblon. However, Golc still had some ability to reduce the proliferation of Pom‐resistant Daudi cells, suggesting that the newer drugs still may have some residual activity. These results are consistent with a previous report, which showed that the proliferation of a panel of Pom‐resistant MM cell lines was either not affected or only minimally affected by Iber [41]. Given that CBIs can function through effector cell immunomodulation (i.e., by increasing the activity of T‐cells and NK‐cells), our findings do not preclude the possibility that these drugs might still be effective in patients who were previously treated with or developed resistance to the older generation CBIs, Pom and Len, through their effect on immune cells. Supporting this notion is the finding that cereblon‐depleted Len/Pom‐resistant MM cell lines were still susceptible to lysis by NK‐cells treated with Iber [41]. Additionally, in a Phase I/II clinical trial, Iber showed clinical activity against relapsed and/or refractory MM, including those refractory to Len or Pom [30, 31], strongly supporting the rationale for investigating these novel CBIs in relapsed and refractory PEL and BL.

In summary, we show that the novel CBIs, Golc and Iber, have substantial activity in PEL and BL, two aggressive NHLs greatly in need of alternative therapeutic interventions. Our observations that these CBIs exert their anticancer effects via multi‐faceted mechanisms and that these activities can be achieved at clinically relevant concentrations make these drugs, especially Golc, attractive candidates for clinical investigation in these malignancies.

## Author Contributions

Conceptualization: Prabha Shrestha, Emma Treco, and Robert Yarchoan. Initial draft preparation: Prabha Shrestha and Emma Treco. Supervision of the study: Robert Yarchoan. Methodology and analysis: Prabha Shrestha and Emma Treco. Study Design: Prabha Shrestha, Emma Treco, David A. Davis, and Robert Yarchoan. Draft revision and editing: Prabha Shrestha, Emma Treco, David A. Davis, and Robert Yarchoan. All authors have reviewed and approved the manuscript.

## Conflicts of Interest

R. Yarchoan reports receiving research support from Celgene (now Bristol Myers Squibb), CTI BioPharma (a Sobi A.B. Company), PDS Biotech, and Janssen Pharmaceuticals through Cooperative Research and Development Agreements (CRADAs) with the National Cancer Institute (NCI). Dr. Yarchoan also reports receiving drugs for clinical trials from Merck, EMD‐Serano, and Eli Lilly and preclinical material from Lentigen Technology through CRADAs or Material Transfer Agreements (MTAs) with the NCI. R. Yarchoan and D.A. Davis are co‐inventors on US Patent 10,001,483 entitled “Methods for the treatment of Kaposi sarcoma or KSHV‐induced lymphoma using immunomodulatory compounds and uses of biomarkers.” An immediate family member of R. Yarchoan is a coinventor on patents or patent applications related to internalization of target receptors, epigenetic analysis, and ephrin tyrosine kinase inhibitors. All rights, title, and interest to these patents have been assigned to the US Department of Health and Human Services; the government conveys a portion of the royalties it receives to its employee inventors under the Federal Technology Transfer Act of 1986 (P.L. 99‐502). The other authors declare no conflicts of interest.

## Supporting information


**Supplementary Figure S1:** Viability of PEL cell lines when cultured with CBIs.
**Supplementary Figure S2:** mRNA levels of KSHV and EBV genes with CBIs.
**Supplementary Figure S3:** Pom does not affect the viability of Pom‐resistant (pomR) cells.
**Supplementary Figure S4:** Cell viability of PEL and BL cell lines at 2 days post‐treatment.
**Supplementary Figure S5:** ICAM‐1 and B7‐2 surface expression in PEL cell lines upon CBI‐treatment.
**Supplementary Figure S6:** Effects of CBIs on MHC‐I in PEL cell lines.
**Supplementary Figure S7:** ICAM‐1 and B7‐2 surface expression in EBV‐positive BL cell lines upon CBI‐treatment.
**Supplementary Figure S8:** Golc and Iber increase MICA in Daudi but not Raji cell line.
**Supplementary Figure S9:** Effect of CBIs on immune surface markers of EBV negative BL cell lines.
**Supplementary Table S1:** List of Primary antibodies used in western blotting.

Supporting File 1

Supporting File 2

## Data Availability

All the data is either included in the manuscript or supplied as a supporting file.
