# Supplementary material for: Novel Cereblon‐Binding Immunomodulators Have Increased Potency Against Gammaherpesvirus‐ Associated Lymphomas In Vitro
Source: J Med Virol. 2025 Aug 6;97(8):e70537. doi: 10.1002/jmv.70537 (PMC13366447; doi:10.1002/jmv.70537)
Supplement: Supplementary file 1 — Supplementary Figure S1: Viability of PEL cell lines when cultured with CBIs. Supplementary Figure S2: mRNA levels of KSHV and EBV genes with CBIs. Supplementary Figure S3: Pom does not affect the viability of Pom‐resistant (pomR) cells. Supplementary Figure S4: Cell viability of PEL and BL cell lines at 2 days post‐treatment. Supplementary Figure S5: ICAM‐1 and B7‐2 surface expression in PEL cell lines upon CBI‐treatment. Supplementary Figure S6: Effects of CBIs on MHC‐I in PEL cell lines. Supplementary Figure S7: ICAM‐1 and B7‐2 surface expression in EBV‐positive BL cell lines upon CBI‐treatment. Supplementary Figure S8: Golc and Iber increase MICA in Daudi but not Raji cell line. Supplementary Figure S9: Effect of CBIs on immune surface markers of EBV negative BL cell lines. Supplementary Table S1: List of Primary antibodies used in western blotting. [file JMV-97-e70537-s003.pdf]

Supplementary Materials and Methods

Real-time quantitative PCR (RT-qPCR). RT-qPCR was performed as described previously (1) on total RNA extracted 2 days post-treatment with CBIs or DMSO control. mRNA expression levels of KSHV and EBV genes were normalized to that of 18S endogenous RNA control and changes in mRNA expression were calculated using ΔΔCt method. Sequences of the primers (5' to 3') are listed below.

LANA	F	GTGACCTTGGCGATGACCTA
	R	CAGGAGATGGAGAATGAGTA
RTA	F	GTCATGTCACCCTTGCGATC
	R	ACGCTTCTTTGAGCTCCTCT
18S	F	GCCCGAAGCGTTTACTTTGA
	R	TCCATTATTCCTAGCTGCGGTATC
EBNA-1	F	CCGCAGATGACCCAGGAGAA
	R	TGGAAACCAGGGAGGCAAAT
EBER-2	F	GGACAGCCGTTGCCCTAGTGG
	R	AGCGGACAAGCCGAATACCCTTC
BMRF-1	F	CGTGCCAATCTTGAGGTTTT
	R	CGGAGGCGTGGTTAAATAAA

Supplementary Table 1

List of Primary antibodies used in western blotting. Proteins were probed either for 1.5 hours at room temperature or overnight at 4^0C.

Antibody	Host	Dilution	Company	Catalog #
alpha TBP [1TBP18]	Mouse	1:1000 in 5% milk in TBST	Abcam	AB818-1001
c-Myc [Y69]	Rabbit	1:10,000 in 5% milk in TBST	Abcam	AB32072-1001
IRF4	Rabbit	1:1000 in 5% milk in TBST	Cell Signaling Technology	4964S
Ikaros/IZKF1	Mouse	1:1000 in 5% milk in TBST	Sigma-Aldrich	SAB4200366
ß-actin	Mouse	1:100,000 in 50% Intercept (TBS) blocking buffer (Licor)	Sigma-Aldrich	A5441
ICAM-1	Mouse	1:1,000 in 50% Intercept (TBS) blocking buffer (Licor)	Invitrogen	60299-1
B7-2	Goat	1:1,000 in 50% Intercept (TBS) blocking buffer (Licor)	R&D	AF-141-NA

Supplementary Figure Legends

Fig S1. Viability of PEL cell lines when cultured with CBIs. (A) KSHV/EBV positive PEL cell lines BC-1 and BC-2 were cultured in the presence of DMSO control or various concentrations of Golc, Iber, or Pom for 5 days. Cell viability was measured using the ATP viability assay (Promega), and relative viability in the presence of CBIs was calculated as a percentage of DMSO control. Error bars represent the standard deviations from 3 (BC-1) or 2 (BC-2) separate experiments, and dose-response curves were plotted using 4PL best fit graphs. Dotted lines represent 50 percent growth inhibition. (B) Absolute IC_{50} values for growth inhibition by each of the CBI in each of the PEL cell line are shown. IC_{50} determinations were made separately from individual experiments and the mean and standard deviation of these IC_{50} values are shown. IC_{50} for Pom-treatment could be determined only from one experiment for each cell line, thus only one value is shown for Pom.

Fig S2. mRNA levels of KSHV and EBV genes with CBIs. Total RNA extracted 2 days post treatment with CBIs or DMSO control were used for qRT-PCR analysis. Expression levels of KSHV and EBV genes relative to control-treatment were calculated after normalization to 18S internal control. Genes measured for KSHV were LANA (latent) and RTA (lytic). Genes measured for EBV were a latent gene EBNA-1, a latent non-coding RNA EBER-2, and a lytic gene BMRF-1. (A) Levels of KSHV genes in BCBL-1 and both KSHV and EBV genes in JSC-1. (B) Levels of EBV genes in EBV+ve BL cell lines Daudi and BL41. Data represent averages from 3 experiments except for JSC-1 with Iber treatment (2 experiments). ND refers to not detected. Statistically significant differences (* $p \leq 0.05$; unpaired, two-tailed, t-test) in changes between Ctrl and CBI-treated cells are shown.

Fig S3. Pom does not affect the viability of Pom-resistant (pomR) cells. PomR PEL cell line, BCBL-1 (A), and BL cell line, Daudi (B), were cultured in the presence of DMSO control or various concentrations of Pom for 5 days. Cell viability was measured using ATP viability assay (Promega) and relative viability in the presence of Pom was calculated as a percentage of DMSO control. Data represents averages ± standard deviations from 4 (BCBL-1) and 3 (Daudi) independent experiments.

Fig S4. Cell viability of PEL and BL cell lines at 2 days post-treatment. Viability of (A) PEL cell lines, BCBL-1 and JSC-1 cells (live cells as a percent of total cells), and (B) BL cell lines, Daudi, Raji, and BL41(EBV+), was assessed 2 days after treatment with DMSO control or CBIs using trypan blue counts. Data shows viability in the presence of CBIs as a percentage of control treatment.

Fig S5. ICAM-1 and B7-2 surface expression in PEL cell lines upon CBI-treatment. (A) Surface levels of ICAM-1 and B7-2 on latent BCBL-1 and JSC-1 cells were measured by flow cytometry after 2 days treatment with various concentrations of Golc/Iber/Pom using PerCP-Cy5.5 labeled antibodies. Experiment was conducted at least 3 times, and one representative experiment with indicated concentrations of the CBIs is shown. Histograms were generated using FlowJo software. (B) BCBL-1 cells were cultured in the presence of DMSO control or 0.01, 0.1, or 1 μM Pom for 2 days prior to measuring the levels of ICAM-1 and B7-2 by flow cytometry. Median fluorescent intensity (MFI) obtained for isotype control was subtracted and fold changes over DMSO control were calculated. Shown are averages from at least 3 independent experiments ± standard deviations. (C) Western blotting was performed on whole cell lysates obtained 3 days-post treatment of BCBL-1 and JSC-1 cell lines and levels of ICAM-1 and B7-2 were measured. Fold changes in ICAM-1 and B7-2 levels induced by CBIs relative to DMSO control after normalization to b-actin (loading control) are shown below the blots.

Fig S6. Effects of CBIs on MHC-I in PEL cell lines. Surface MHC-I levels were measured by flow cytometry on latent BCBL-1 (A) and JSC-1 (B) cells after 2 (BCBL-1) or 3 (JSC-1) days treatment with DMSO control or indicated concentrations of the CBIs. Fold changes in MFI over DMSO control were calculated and averages from at least 3 independent experiments ± standard deviations are shown. (C) Surface MHC-I levels were measured after lytic induction of BCBL-1 and JSC-1 cells with NaB (BCBL-1) or TPA (JSC-1). Cells were pretreated with DMSO control or CBIs for 24 hours (BCBL-1) or 48 hours (JSC-1) and then treated with NaB or TPA for another 24 hours prior to measuring surface MHC-I levels. Experiment was conducted at least 3 times, and one representative experiment with indicated concentrations of the CBIs is shown. Histograms were generated using FlowJo software.

Fig S7. ICAM-1 and B7-2 surface expression in EBV-positive BL cell lines upon CBI-treatment. (A) Surface levels of ICAM-1 and B7-2 on Daudi, Raji, and BL41(EBV+) cells were measured by flow cytometry after 2 days treatment with various concentrations of Golc/Iber/Pom using PerCP-Cy5.5 labeled antibodies. Experiment was conducted at least 3 times, and one representative experiment with indicated concentrations of the CBIs is shown. Histograms were generated using flowJo software. (B) Daudi cells were cultured in the presence of DMSO control or 0.01, 0.1, or 1 μM Pom for 2 days prior to measuring the levels of ICAM-1 and B7-2 by flow cytometry. Median fluorescent intensity (MFI) obtained for isotype control was subtracted and fold changes over DMSO control were calculated. Shown are averages from at least 3 independent experiments ± standard deviations. (C) Western blotting was performed on whole cell lysates obtained 3 days-post treatment of BL cell lines and levels of ICAM-1 and B7-2 were measured. Fold changes in ICAM-1 and B7-2 levels induced by CBIs relative to DMSO control after normalization to b-actin (loading control) are shown below the blots.

Fig S8. Golc and Iber increase MICA in Daudi but not Raji cell line. BL cell lines, Daudi(A) and Raji (B), were cultured for 2 days with DMSO control or CBIs, and levels of MICA was measured using flow cytometry. Median fluorescent intensity (MFI) obtained for isotype control was subtracted and fold changes over DMSO control were calculated. Shown are averages from 3 independent experiments ± standard deviations.

Fig S9. Effect of CBIs on immune surface markers of EBV negative BL cell lines. BL41(EBV-) and CA46 cell lines were cultured for 2 days with DMSO control or CBIs, and levels of ICAM-1 (A) and B7-2 (B) were measured using flow cytometry. Median fluorescent intensity (MFI) obtained for isotype control was subtracted and fold changes over DMSO control were calculated. Shown are averages from 3 independent experiments ± standard deviations. Statistically significant differences (* $p \leq 0.05$ ** $p \leq 0.01$; unpaired, two-tailed, t-test) in fold changes between Ctrl and CBI-treated cells are shown.

References

1. Davis DA, Mishra S, Anagho HA, Aisabor AI, Shrestha P, Wang V, et al. Restoration of immune surface molecules in Kaposi sarcoma-associated herpes virus infected cells by lenalidomide and pomalidomide. Oncotarget. 2017;8(31):50342-58.

Supplementary Figures

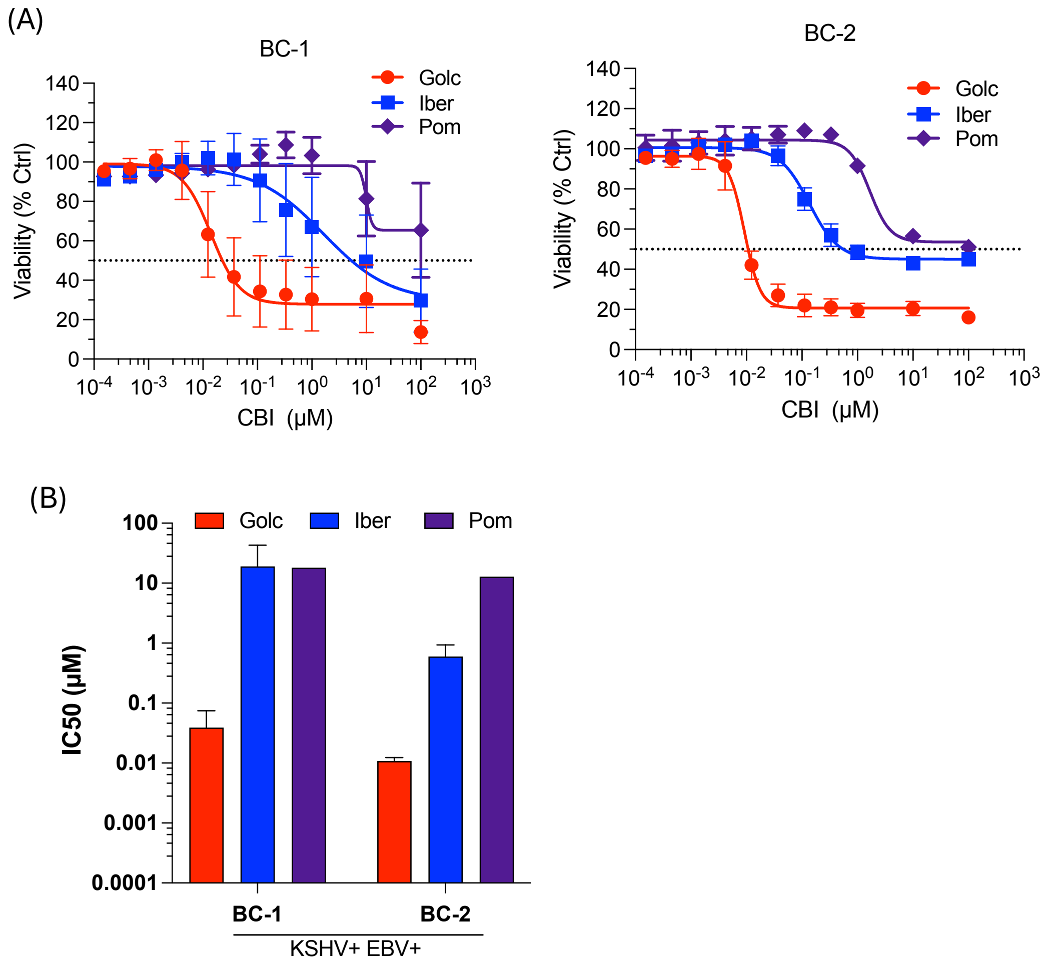

Fig S1

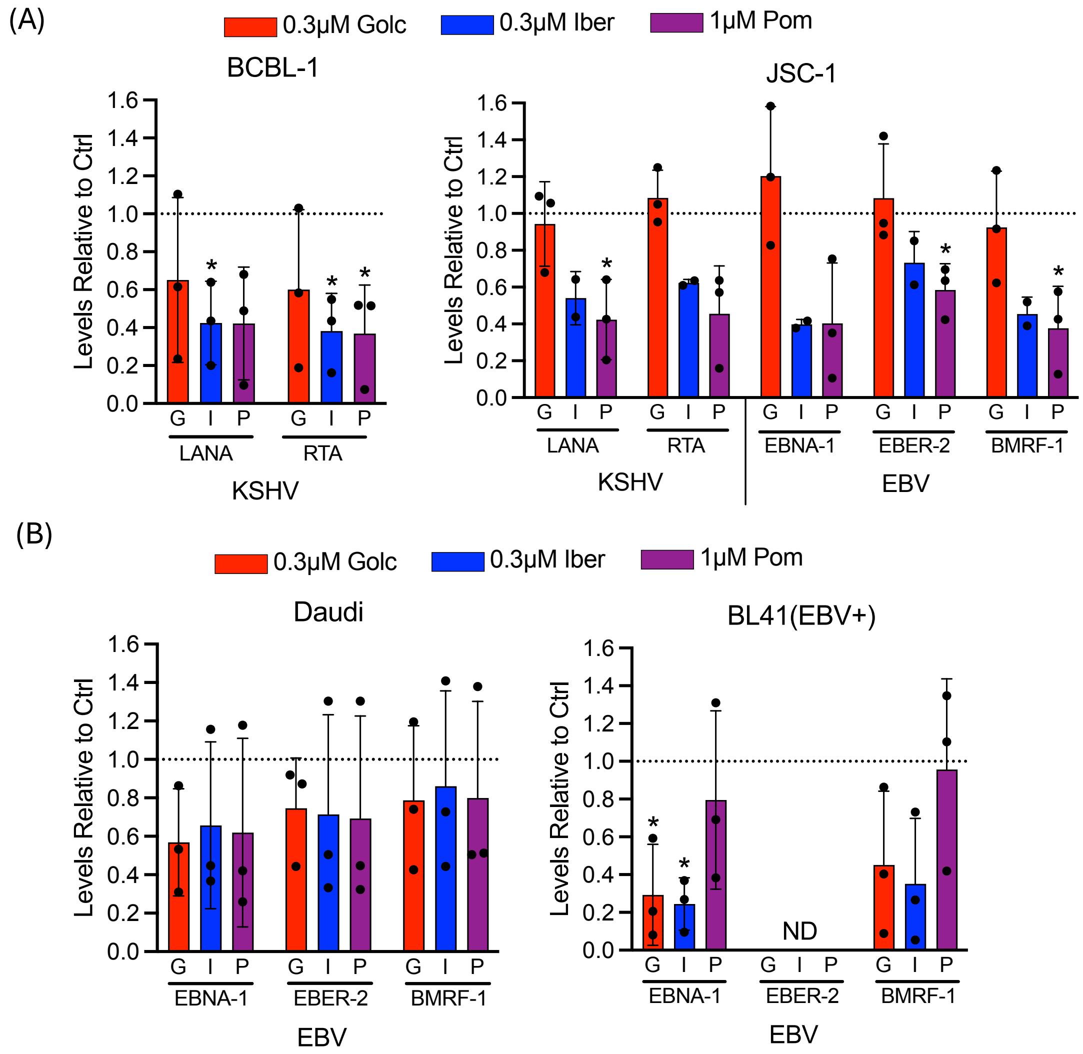

(A)
0.3μM Golc
0.3μM Iber
1μM Pom
BCBL-1
JSC-1
Levels Relative to Ctrl
1.6
1.4
1.2
1.0
0.8
0.6
0.4
0.2
0.0
G I P
LANA
RTA
EBNA-1
EBER-2
BMRF-1
KSHV
EBV
(B)
Daudi
BL41(EBV+)
ND

Fig S2

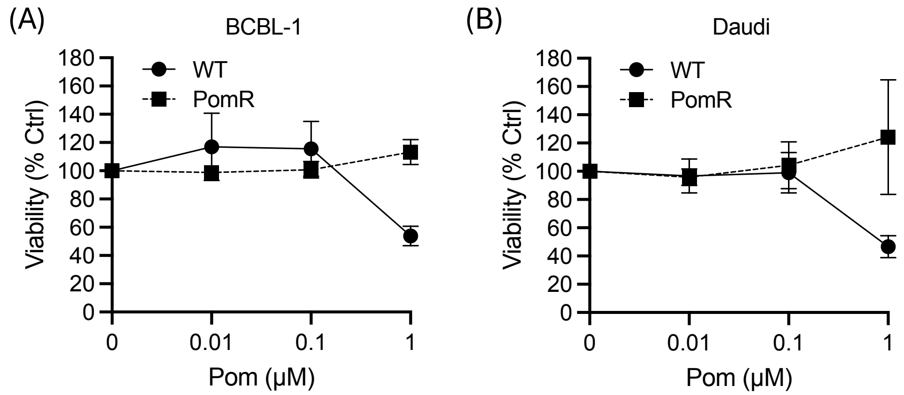
(A)
BCBL-1
WT
PomR
Viability (% Ctrl)
180
160
140
120
100
80
60
40
20
0
0
0.01
0.1
1
Pom (µM)
(B)
Daudi
WT
PomR
Viability (% Ctrl)
180
160
140
120
100
80
60
40
20
0
0
0.01
0.1
1
Pom (µM)

Fig S3

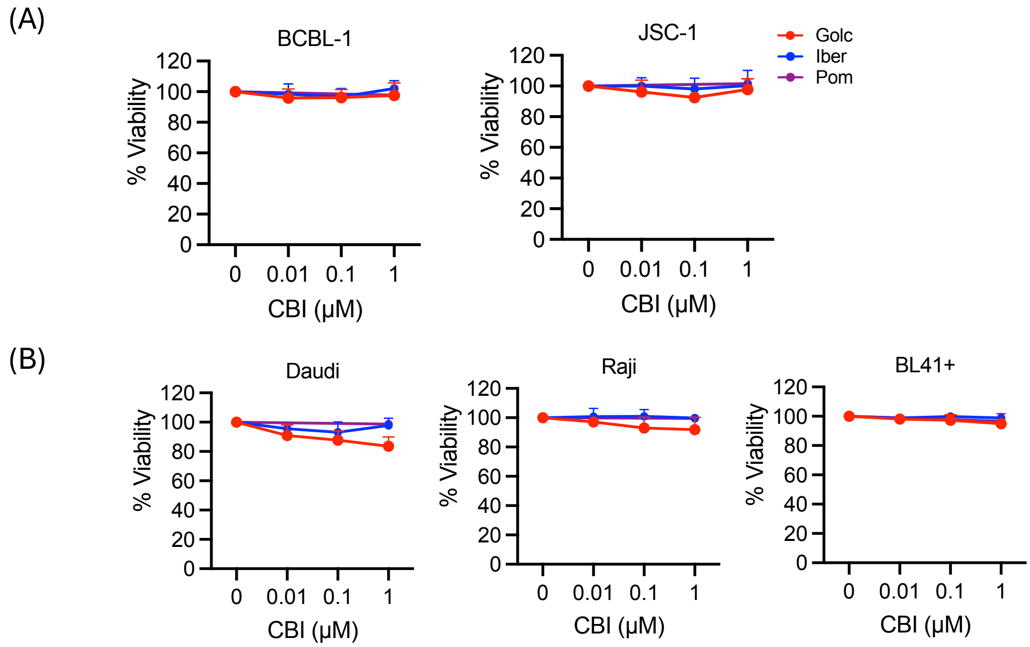
(A)
BCBL-1
JSC-1
Golc
Iber
Pom
% Viability
120
100
80
60
40
20
0
0
0.01
0.1
1
CBI (μM)
(B)
Daudi
Raji
BL41+

Fig S4

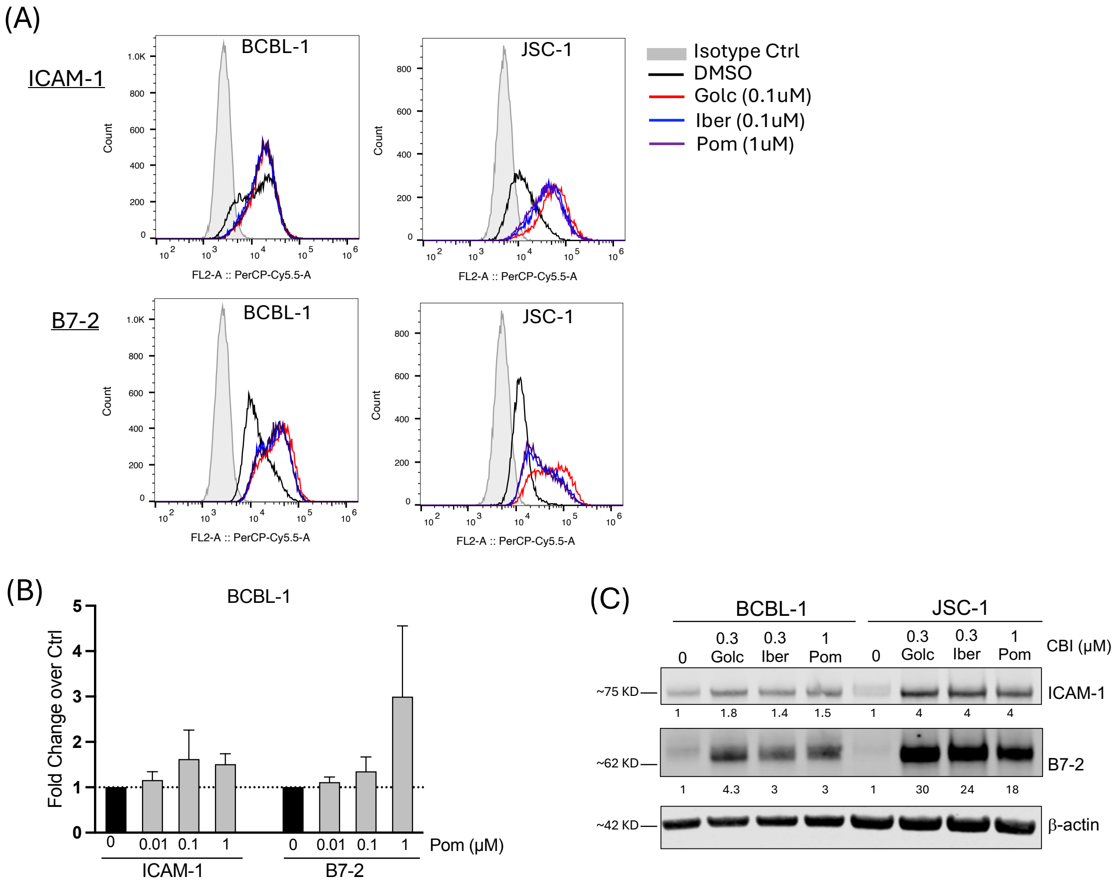
(A)
ICAM-1
B7-2
BCBL-1
JSC-1
Count
FL2-A :: PerCP-Cy5.5-A
Isotype Ctrl
DMSO
Golc (0.1uM)
Iber (0.1uM)
Pom (1uM)
(B)
BCBL-1
Fold Change over Ctrl
Pom (μM)
ICAM-1
B7-2
(C)
BCBL-1
JSC-1
CBI (μM)
0
0.3 Golc
0.3 Iber
1 Pom
~75 KD
~62 KD
~42 KD
ICAM-1
B7-2
β-actin
1 1.8 1.4 1.5 1 4 4 4
1 4.3 3 3 1 30 24 18

Fig S5

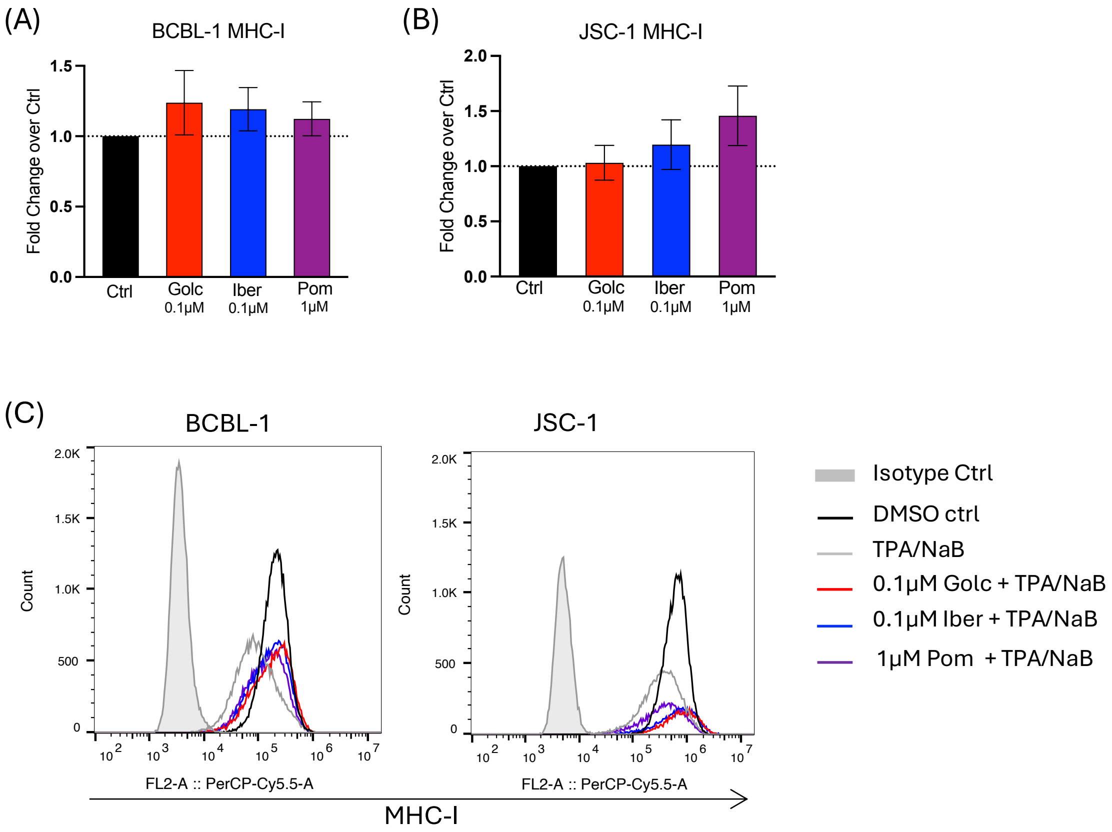

(A)
BCBL-1 MHC-I
Fold Change over Ctrl
1.5
1.0
0.5
0.0
Ctrl
Golc 0.1μM
Iber 0.1μM
Pom 1μM
(B)
JSC-1 MHC-I
Fold Change over Ctrl
2.0
1.5
1.0
0.5
0.0
Ctrl
Golc 0.1μM
Iber 0.1μM
Pom 1μM
(C)
BCBL-1
JSC-1
Count
2.0K
1.5K
1.0K
500
0
FL2-A :: PerCP-Cy5.5-A
MHC-I
Isotype Ctrl
DMSO ctrl
TPA/NaB
0.1μM Golc + TPA/NaB
0.1μM Iber + TPA/NaB
1μM Pom + TPA/NaB

Fig S6

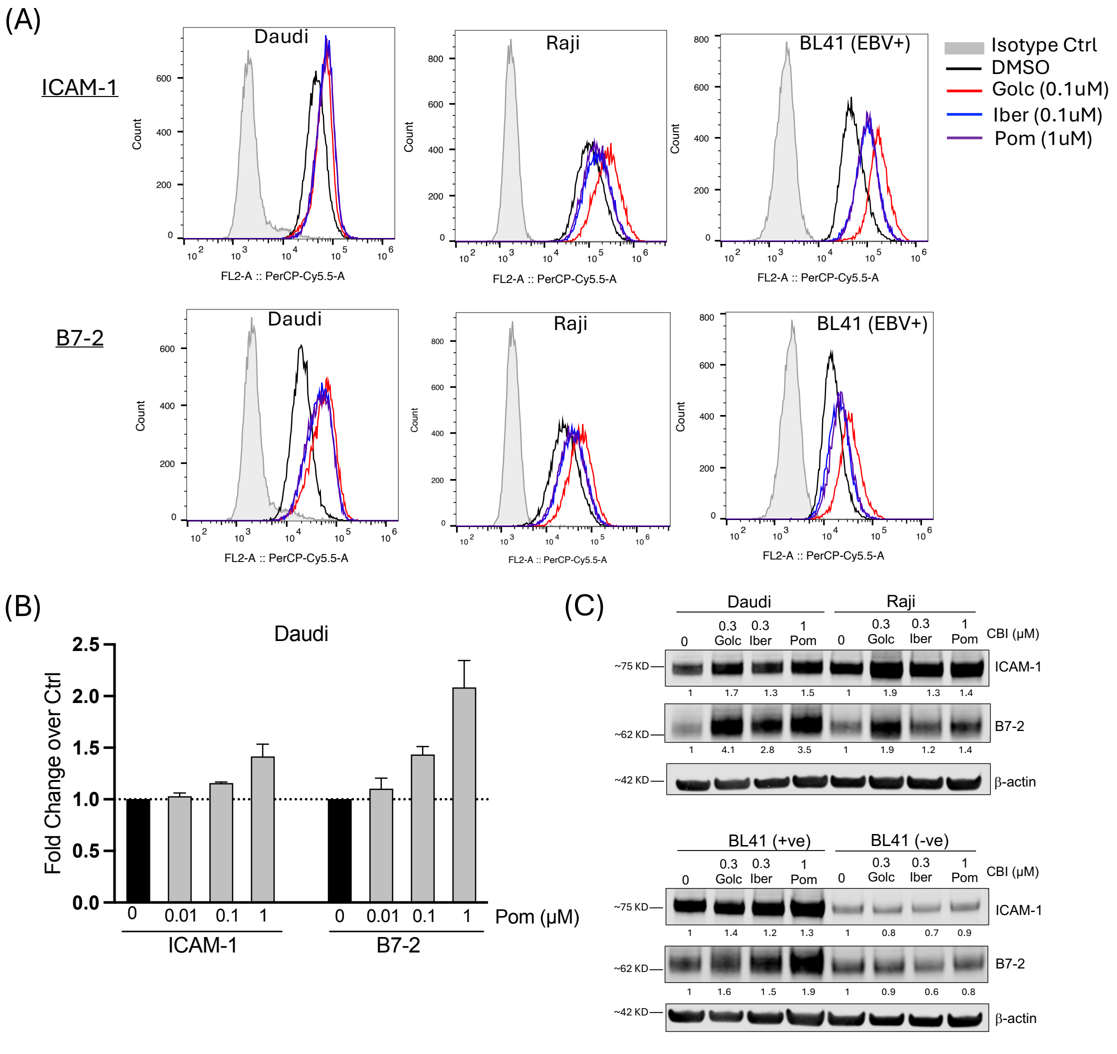
(A)
ICAM-1
B7-2
Daudi
Raji
BL41 (EBV+)
Isotype Ctrl
DMSO
Golc (0.1uM)
Iber (0.1uM)
Pom (1uM)
Count
FL2-A :: PerCP-Cy5.5-A
(B)
Daudi
Fold Change over Ctrl
0 0.01 0.1 1 0 0.01 0.1 1 Pom (μM)
ICAM-1
B7-2
(C)
Daudi
Raji
0 0.3 Golc 0.3 Iber 1 Pom 0 0.3 Golc 0.3 Iber 1 Pom CBI (μM)
~75 KD
ICAM-1
1 1.7 1.3 1.5 1 1.9 1.3 1.4
~62 KD
B7-2
1 4.1 2.8 3.5 1 1.9 1.2 1.4
~42 KD
β-actin
BL41 (+ve)
BL41 (-ve)
0 0.3 Golc 0.3 Iber 1 Pom 0 0.3 Golc 0.3 Iber 1 Pom CBI (μM)
~75 KD
ICAM-1
1 1.4 1.2 1.3 1 0.8 0.7 0.9
~62 KD
B7-2
1 1.6 1.5 1.9 1 0.9 0.6 0.8
~42 KD
β-actin

Fig S7

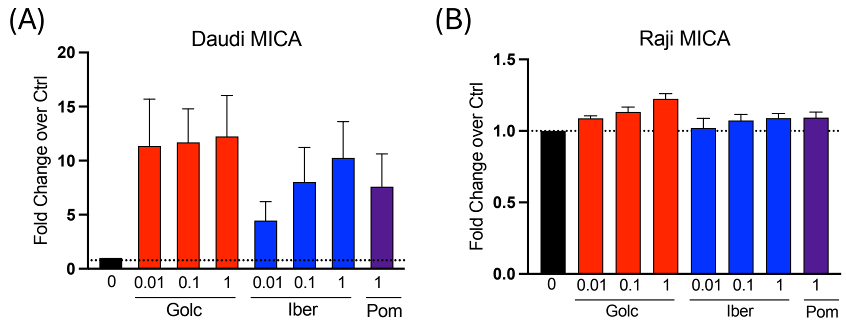
(A)
Daudi MICA
Fold Change over Ctrl
20
15
10
5
0
0
0.01
0.1
1
0.01
0.1
1
1
Golc
Iber
Pom
(B)
Raji MICA
Fold Change over Ctrl
1.5
1.0
0.5
0.0
0
0.01
0.1
1
0.01
0.1
1
1
Golc
Iber
Pom

Fig S8

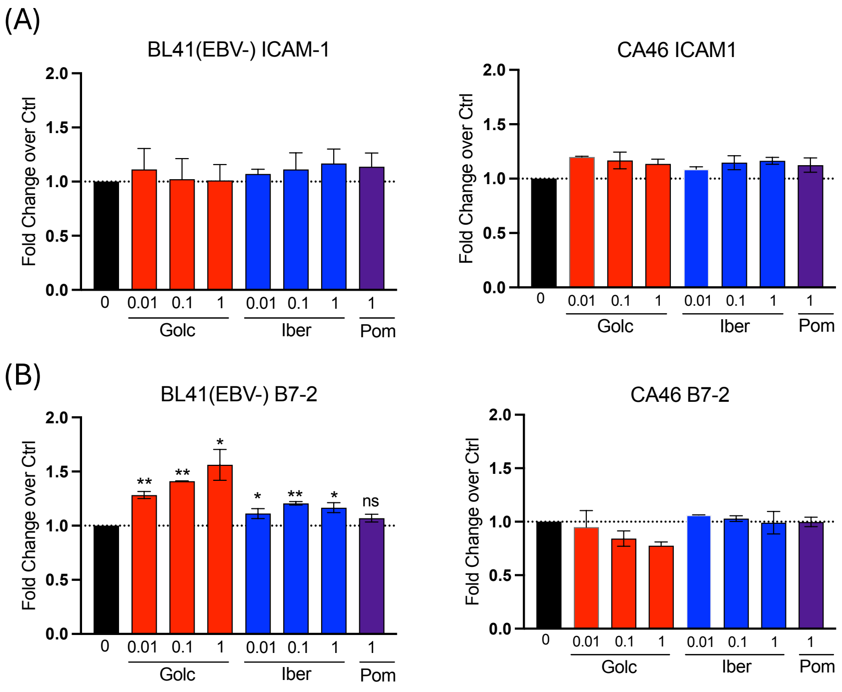
(A)
BL41(EBV-) ICAM-1
CA46 ICAM1
Fold Change over Ctrl
2.0
1.5
1.0
0.5
0.0
0
0.01
0.1
1
Golc
Iber
Pom
(B)
BL41(EBV-) B7-2
CA46 B7-2
**
*
ns

Fig S9
