# Supplementary material for: Novel Cereblon‐Binding Immunomodulators Have Increased Potency Against Gammaherpesvirus‐ Associated Lymphomas In Vitro
Source: J Med Virol. 2025 Aug 6;97(8):e70537. doi: 10.1002/jmv.70537 (PMC13366447; doi:10.1002/jmv.70537)

Supplemental Material: Full Westerns

Fig 3A: JSC-1

Order of antibodies:

1. Ms anti-IKZF1(Ikaros) and Rb anti-cMyc
2. Blot stripped with LICOR Stripping Buffer
3. Ms anti-TBP and Rb anti-IRF4

Experiment 1

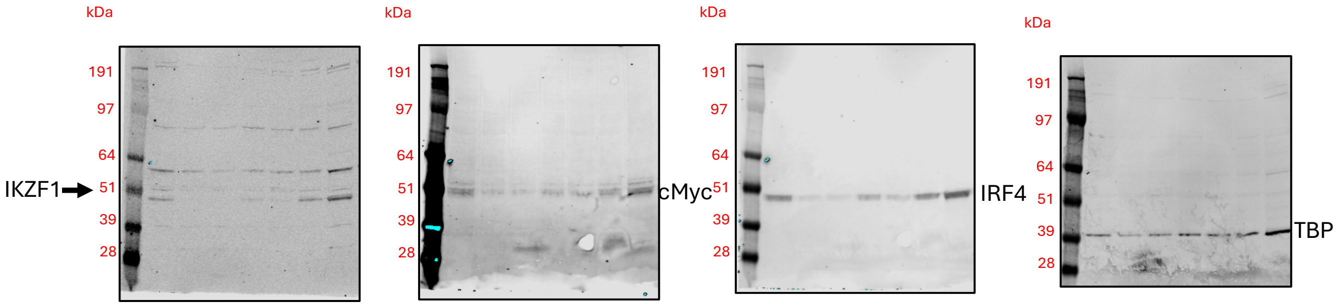

Experiment 2: Representative blot used

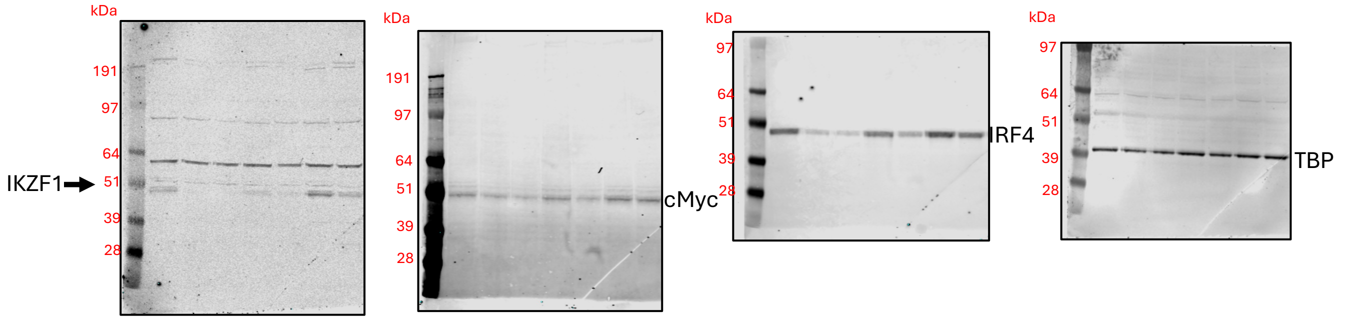

Experiment 3

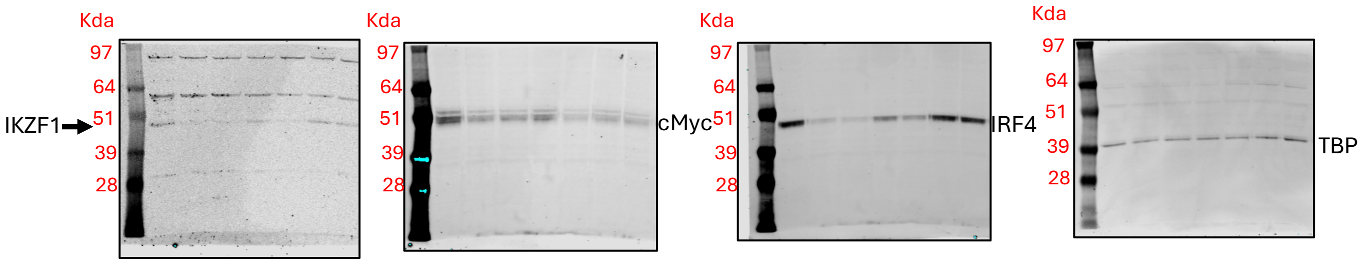

Fig 3B: BCBL-1

Order of antibodies:

1. Ms anti-IKZF1(Ikaros) and Rb anti-cMyc
2. Blot stripped with LICOR Stripping Buffer
3. Ms anti-TBP and Rb anti-IRF4

Experiment 1

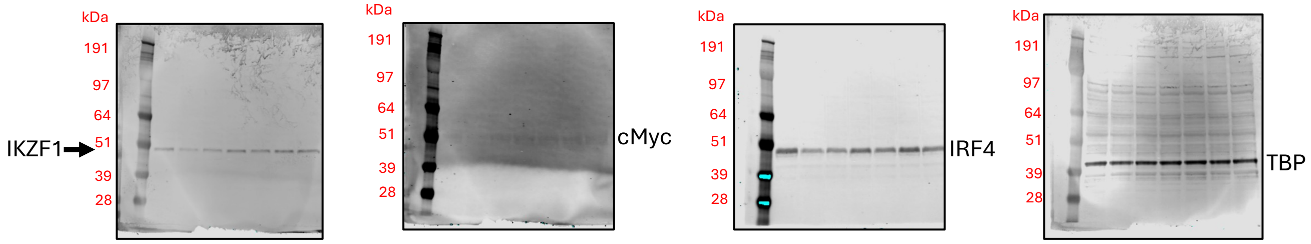

Experiment 2

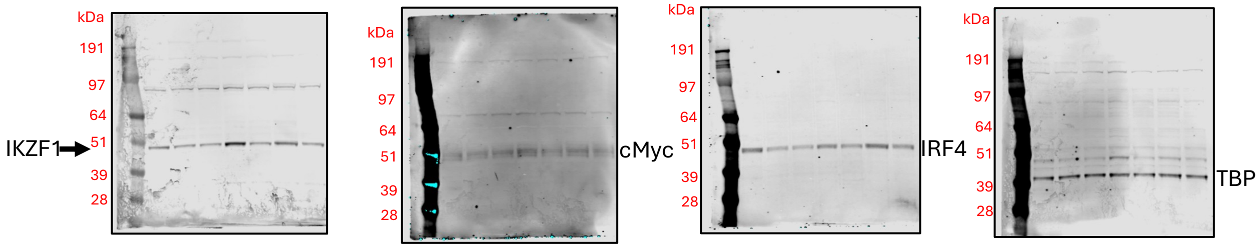

Experiment 3: used as representative blot

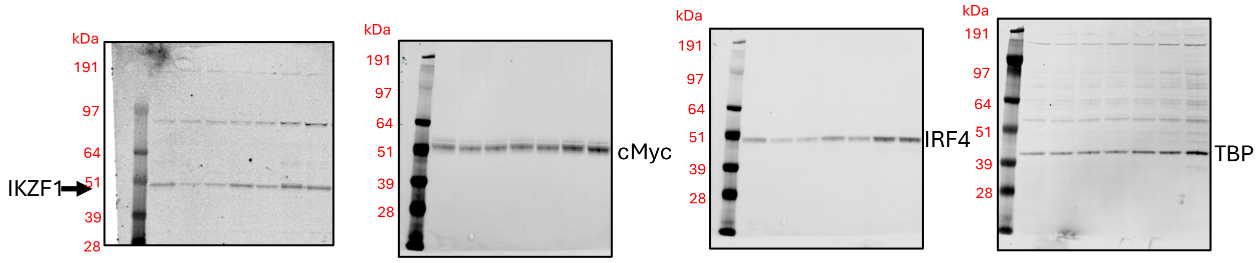

Fig 3C: Daudi

Order of antibodies:

1. Ms anti-IKZF1(Ikaros) and Rb anti-cMyc
2. Blot stripped with LICOR Stripping Buffer
3. Ms anti-TBP and Rb anti-IRF4

* Note incomplete stripping of IKZF1, which can be detected in TBP blot

<u>Experiment 1: Used as representative blot</u>

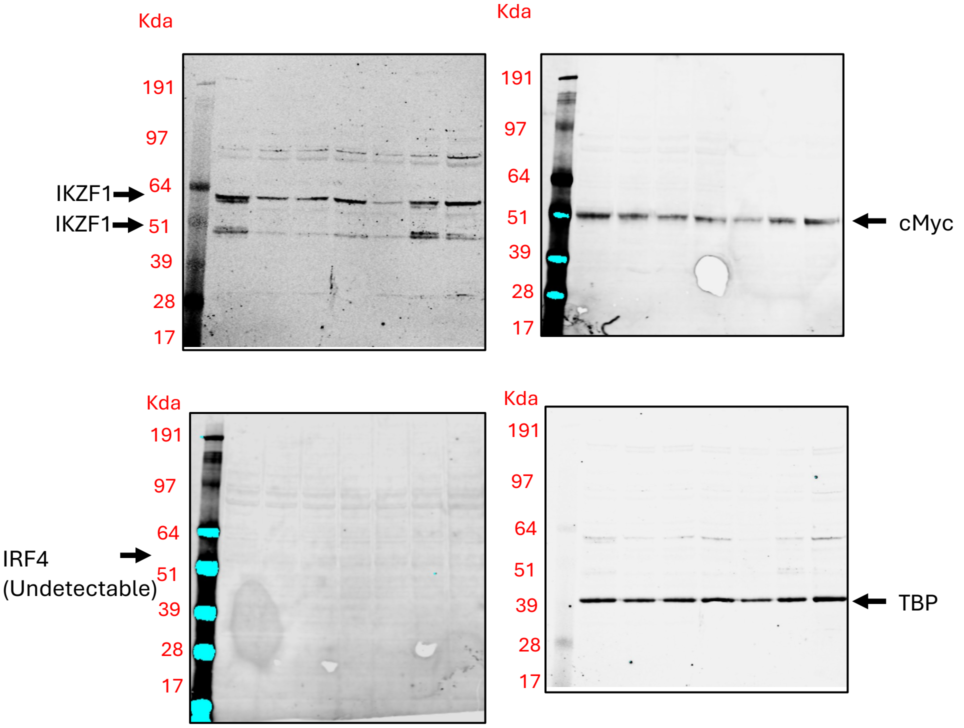

<u>Experiment 2</u>

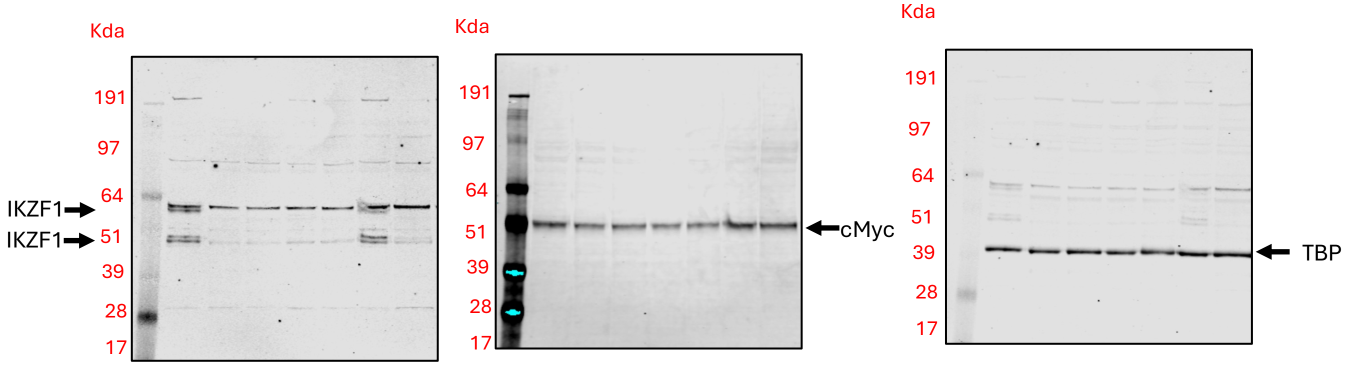

Fig 3D: BL41+/-

Order of antibodies:

1. Ms anti-IKZF1(Ikaros) and Rb anti-cMyc
2. Blot stripped with LICOR Stripping Buffer
3. Ms anti-TBP and Rb anti-IRF4

* Note incomplete stripping of IKZF1, which can be detected in TBP blot

Experiment 1

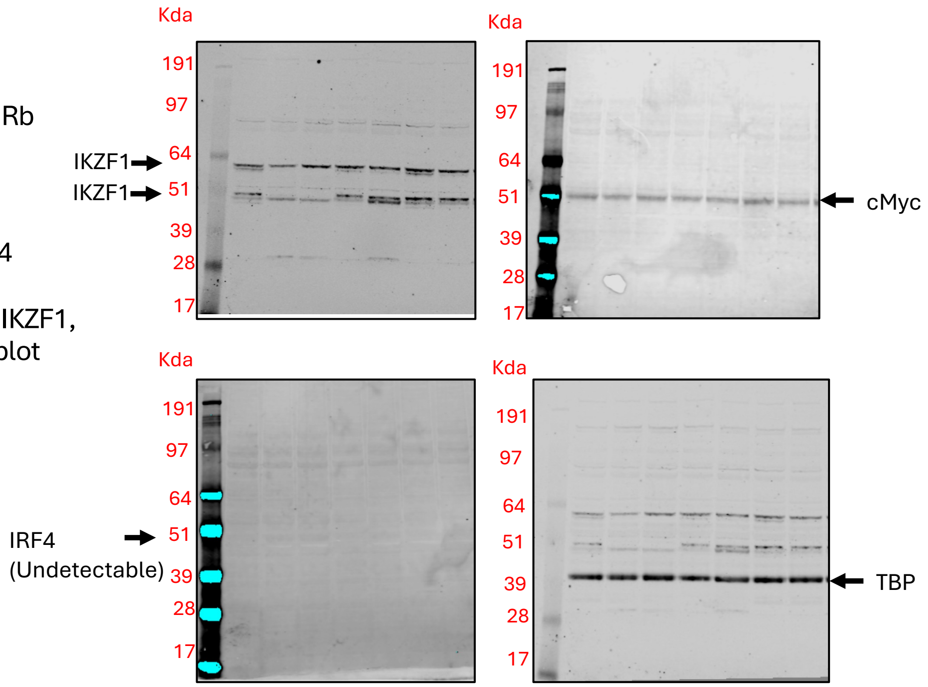

Experiment 2: Used as representative blot

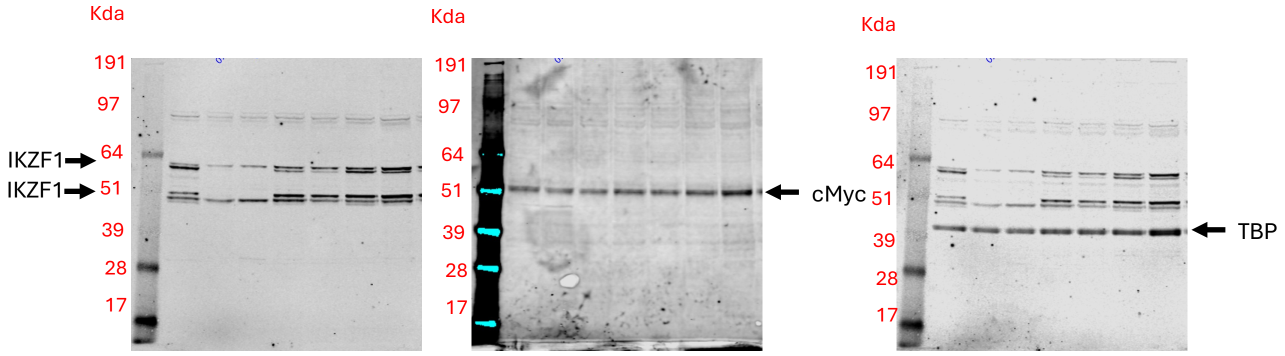

Experiment 3

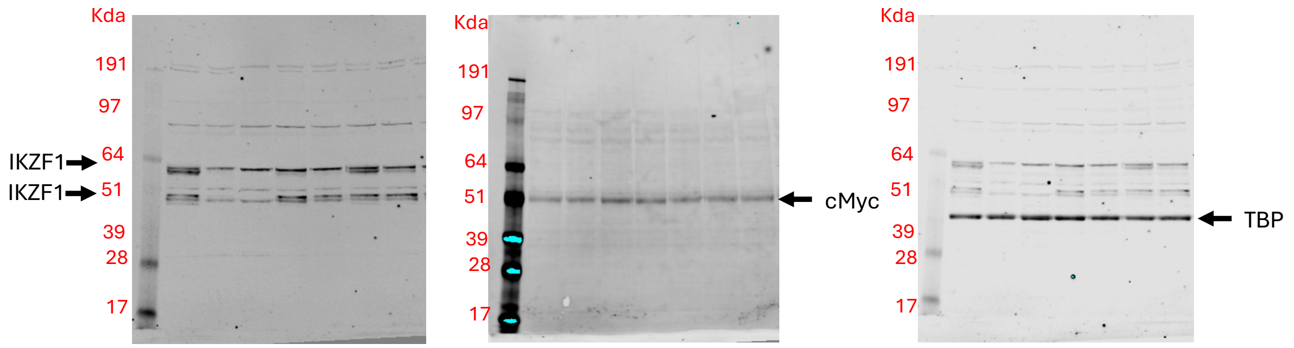

Fig S5C: PEL cell lines (BCBL-1 and JSC-1)

Membrane cut at ~49 KD to separate the upper and lower blots

1. Upper blot: Ms anti-ICAM-1 and Rb anti-B7-2
2. Lower blot: Ms anti β-actin

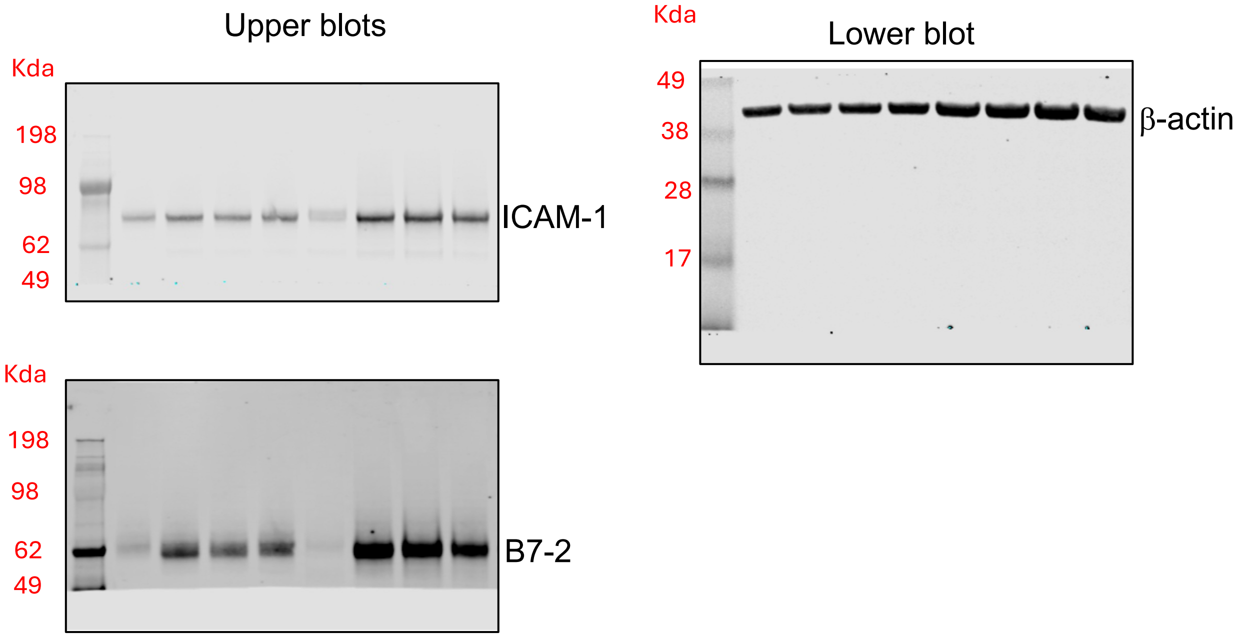

Fig S7C: BL cell lines

Membranes cut above 49 KD to separate the upper and lower blots

1. Upper blot: Ms anti-ICAM-1 and Rb anti-B7-2
2. Lower blot: Ms anti β-actin

Daudi and Raji

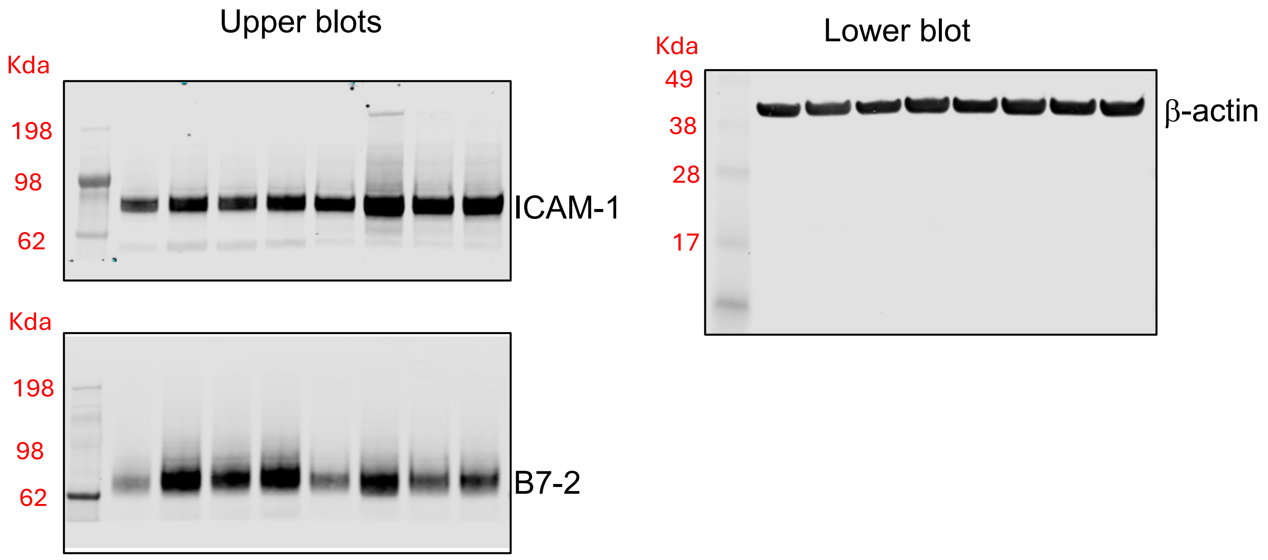

Bl41(+) and (-)

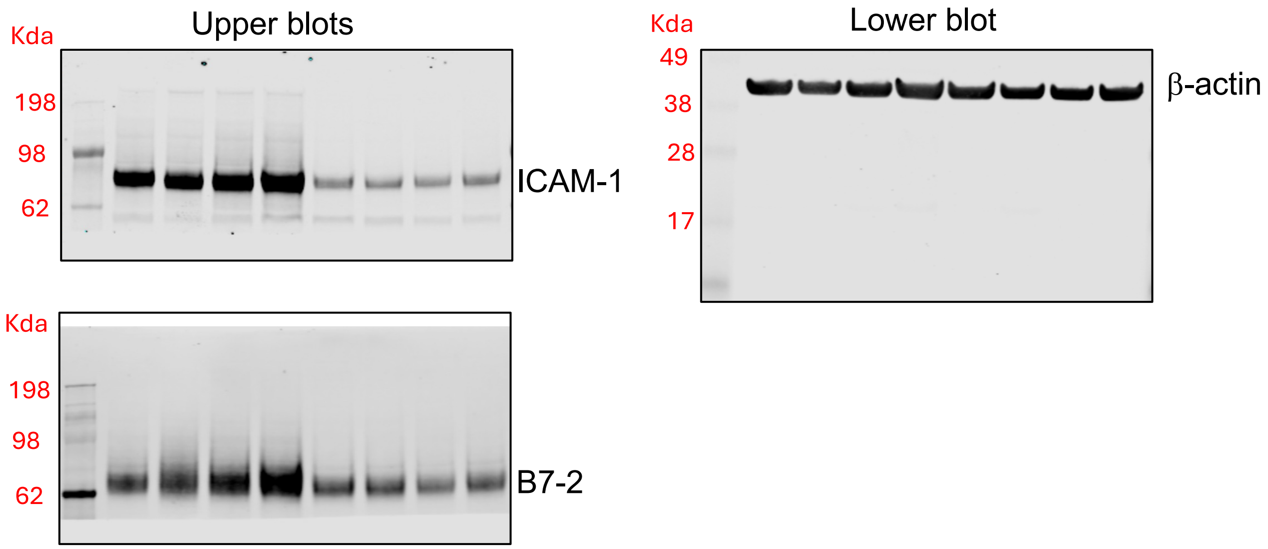

Supplement: Supplementary file 2 — Supporting File 1 [file JMV-97-e70537-s001.pdf]
